# On the Structural and Biological Effects of Hydroxyapatite and Gold Nano-Scale Particles in Poly(Vinylidene Fluoride) Smart Scaffolds for Bone and Neural Tissue Engineering

**DOI:** 10.3390/molecules30051041

**Published:** 2025-02-25

**Authors:** Angelika Zaszczyńska, Marzena Zychowicz, Dorota Kołbuk, Piotr Denis, Arkadiusz Gradys, Paweł Ł. Sajkiewicz

**Affiliations:** 1 Institute of Fundamental Technological Research, Polish Academy of Sciences, Pawinskiego 5B, 02-106 Warsaw, Poland; azasz@ippt.pan.pl (A.Z.); dkolbuk@ippt.pan.pl (D.K.); pdenis@ippt.pan.pl (P.D.); 2 Department of Stem Cell Bioengineering, Mossakowski Medical Research Institute, Polish Academy of Sciences, Pawinskiego 5, 02-106 Warsaw, Poland; mzychowicz@imdik.pan.pl

**Keywords:** scaffolds, tissue engineering, bone tissue engineering, smart medicine, biodegradable polymers, regenerative medicine, poly(vinylidene fluoride)

## Abstract

Piezoelectric materials, due to their ability to generate an electric charge in response to mechanical deformation, are becoming increasingly attractive in the engineering of bone and neural tissues. This manuscript reports the effects of the addition of nanohydroxyapatite (nHA), introduction of gold nanoparticles (AuNPs) via sonochemical coating, and collector rotation speed on the formation of electroactive phases and biological properties in electrospun nanofiber scaffolds consisting of poly(vinylidene fluoride) (PVDF). FTIR, WAXS, DSC, and SEM results indicate that introduction of nHA increases the content of electroactive phases and fiber alignment. The collector rotational speed increases not only the fiber alignment but also the content of electroactive phases in PVDF and PVDF/nHA fibers. Increased fiber orientation and introduction of each of additives resulted in increased SFE and water uptake. In vitro tests conducted on MG-63 and hiPSC-NSC cells showed increased adhesion and cell proliferation. The results indicate that PVDF-based composites with nHA and AuNPs are promising candidates for the development of advanced scaffolds for bone and neural tissue engineering applications, combining electrical functionality and biological activity to support tissue regeneration.

## 1. Introduction

In recent times, nanoscale materials, especially Smart Materials (SM), have the potential to enhance tissue engineering and stem cell therapy [1,2]. Tissue engineering presents an exciting avenue for the development of functional constructs that mimic the structural organization of native tissues, aiming to enhance or replace biological functions, thus potentially obviating the need for organ transplantation. In tissue engineering, the main goal is successful formation of three-dimensional (3D) tissues, which may be achieved by seeding of the biological cells into specially designed manufactured scaffolds [3]. The ideal scaffold for tissue engineering applications should perfectly imitate the functional properties of the extracellular matrix (ECM) of the target tissues for regeneration [4]. However, the intricate chemical and architectural composition of the bone and neural systems pose additional challenges to engineering tissue, necessitating specific criteria in scaffold design, especially in bone and neural tissue engineering.

**Figure 1:**
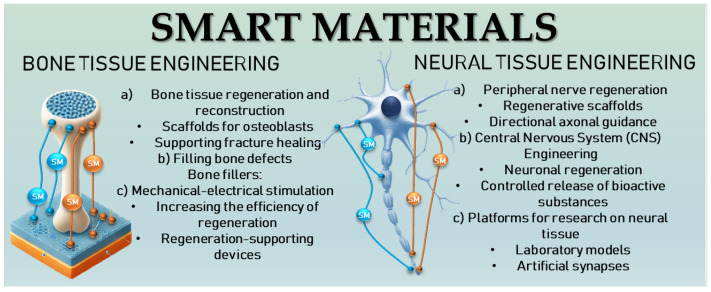
Schematic illustration of electroactive biomaterials for bone and neural tissue regeneration.

One of the responses to such requirements is the use of SM, among which poly(vinylidene fluoride) (PVDF) is very well-known due to its high piezoelectric properties [5]. In this context, various approaches, such as conductive polymers, aligned polymers, and both exogenous and endogenous electrical stimulation, have demonstrated promising outcomes [6]. Figure 1 shows schematic illustration of SM for bone and neural tissue applications.

PVDF is a synthetic polymer known for its high piezoelectric properties and electrospinning ability [7]. PVDF exhibits five distinct crystalline phases, named as, α, β, γ, δ, and ε [8]. Among these, the piezoelectric phases (β, γ, δ) are characterized by a polar crystal structure, where the dipoles align in parallel, resulting in a non-zero dipole moment [9]. The β-phase is particularly significant due to its outstanding piezoelectric, pyroelectric, and ferroelectric properties [10]. In this phase, all dipole moments align in a single direction, producing the strongest piezoelectric response. Polar γ-phase has lower piezoelectricity compared to β-phase, this is due to the presence of *gauche* bond in C-C repeating unit [11].

This thermoplastic polymer has high chemical resistance, and biocompatibility, rendering it indispensable in various electro-optical [12], electromechanical [13], and biomedical applications [14]. Piezoelectric PVDF holds significant promise for advanced applications in cell biology and tissue engineering, particularly in piezoelectric sensing and actuation of cellular activities [15]. Notably, PVDF and its copolymers have emerged as highly promising materials in the realm of biological and biomedical applications [16]. These materials lend themselves readily to processing, yielding well-defined nanofibers that find application in cell culture. PVDF is a non-toxic material with great potential for applications. However, it is important to control the production and modification processes to avoid introducing harmful additives or by-products that could negatively affect the safety of biomedical applications [17]. Given the burgeoning interest in tissue engineering scaffolds with piezoelectric capabilities, PVDF assumes a prime position as an excellent candidate for fabricating bioscaffolds endowed with piezoelectric properties. While the piezoelectric properties of PVDF hold potential applications for neuronal and osteogenic cell outgrowth, thorough demonstrations of this aspect remain rare in the current literature [18,19].

Piezoelectric materials have the unique ability to generate electric charges in response to mechanical deformation, which is particularly useful in the regeneration of neural tissue [20]. In nerve regeneration, electrical signals play a key role because they promote the growth, guidance, and differentiation of nerve cells [21]. Electrical signals generated by piezoelectric materials can activate ion channels in nerve cell membranes, which in turn promotes the release of growth factors and other cellular signals necessary for regeneration. This can lead to increased proliferation and differentiation of nerve cells [22].

Additionally, piezoelectricity is found in bones [23]. Piezoelectric materials play an important role in the regeneration of bone tissue due to their ability to generate electric charges under the influence of mechanical deformations [24]. Bone tissue, similar to nervous tissue, responds to electrical signals that can support regenerative processes. Electrical signals can activate osteoblasts, promoting their proliferation (increase in cell number) and differentiation (transformation into mature bone cells). Increased osteoblast activity leads to faster formation of new bone tissue [25].

Scientists have long been interested in the use of nanohydroxyapatite (nHA) in bone tissue engineering due to its chemical similarity to the natural bone matrix. In the study conducted by Yang et al. [26], nHA was incorporated into poly(lactide-co-glycolide) (PLGA) to enhance the bioactivity of the scaffolds. Their study showed that these composites promoted adhesion, proliferation, and increased expression of osteogenic-related genes, suggesting their potential for use in bone regeneration. Other studies, such as those conducted by Akbari et al. [27], focused on piezoelectric PVDF/PCL/HA composites. They demonstrated that these composites could generate electrical pulses in response to mechanical stress, which could stimulate bone regeneration via mechanotransduction. The results showed that the piezoelectric properties of PVDF combined with the bioactivity of nHA could accelerate the bone healing process. Furthermore, gold nanoparticles (AuNPs) have been widely studied due to their excellent conductive properties and biocompatibility [28]. For example, Motamedi et al. [6] studied PVDF and gold nanoparticle composites, showing that their combination can improve the electrical conductivity of scaffolds, which may be suitable for use as a scaffold in neural tissue engineering. Other studies have also been conducted on electrospun PVDF fibers [29] with additives [30] but without orientation. Still, there are only a few studies on aligned fibers and their effects on the material structure and biological properties. To the authors’ knowledge, studies on the orientation of piezoelectric nanofibrous scaffolds with additives for bone and neural cell regeneration applications are lacking in the literature. All these studies emphasize the growing interest and potential of piezoelectric PVDF, nHA, and AuNPs composites in bone and neural tissue regeneration, indicating their innovativeness and multifaceted impact on developing advanced biomedical scaffolds.

Therefore, our goal was to design piezoelectric scaffolds with random and aligned fiber orientation. Studying various nanofiber orientations and their influence on properties is a new approach that can lead to optimizing the functionality of these materials in multi-biomedical applications. Oriented nanofibers can better simulate the structure of natural tissues and induce the formation of piezoelectric phases, which can be crucial for effective regeneration. On the other hand, the addition of piezoelectric nHA and AuNPs can also increase piezoelectric phase content, material-cell interactions, promoting osseointegration, and can support repair processes in nerve cells. In the context of current advances in the field of SM and their importance for tissue engineering applications, selecting the most optimal method for manufacturing PVDF fibers with strong piezoelectric response is crucial to increase the efficiency of these materials applications while reducing production costs.

## 2. Results and Discussion

### 2.1. Morphology Analysis

**Figure 2:**
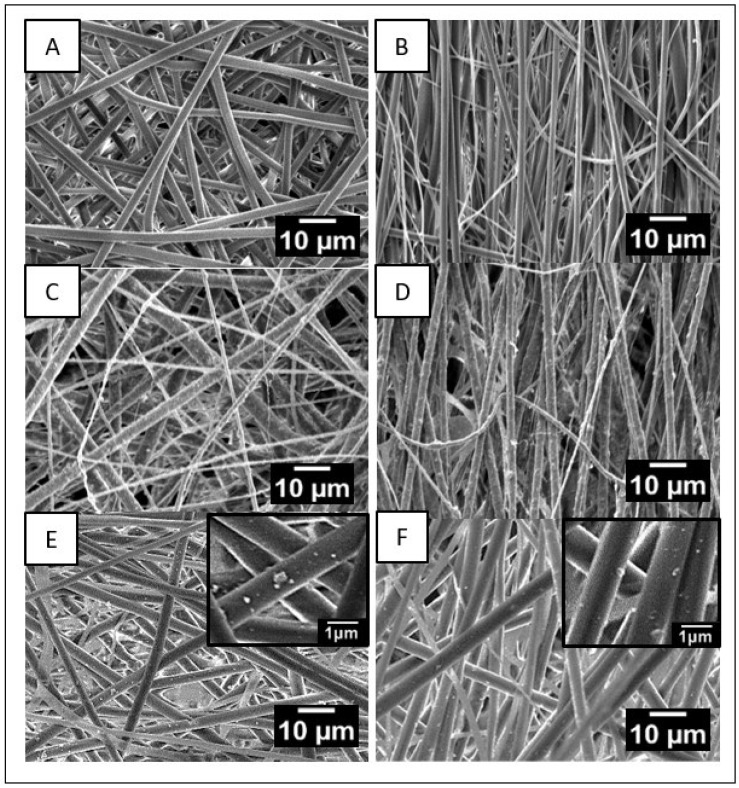
SEM micrographs of (**A**) PVDF_R, (**B**) PVDF_A, (**C**) PVDF/nHA_R, and (**D**) PVDF/nHA_A, (**E**) PVDF/AU_R, (**F**) PVDF/AU_A nanofibrous scaffolds.

**Figure 3:**
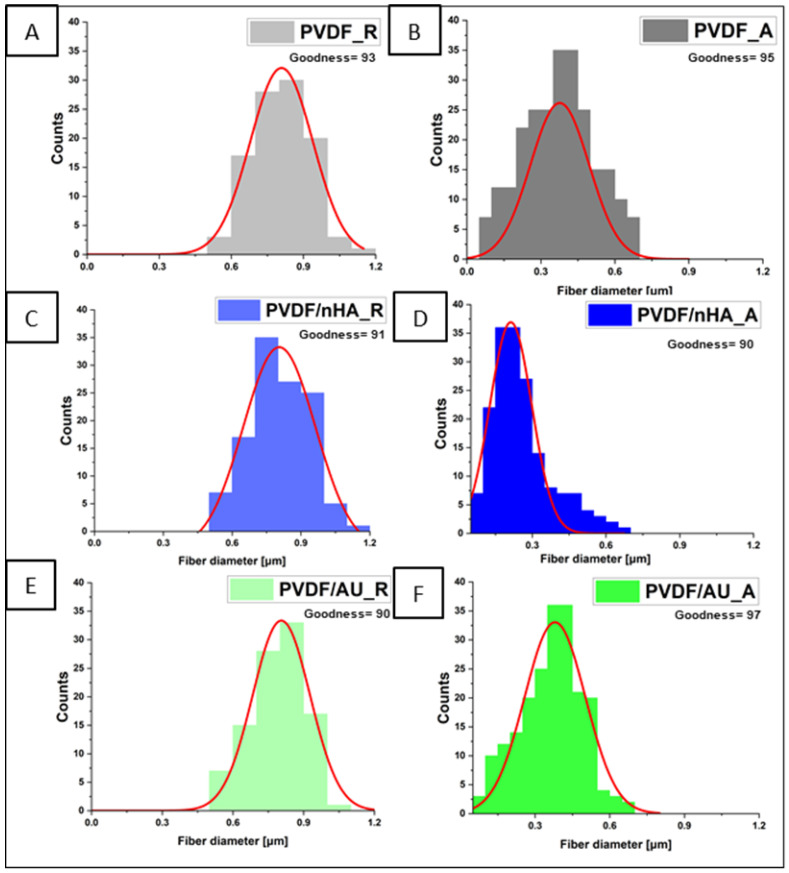
Diameter distributions approximated with Gaussian function of nanofibrous scaffolds (**A**) PVDF_R, (**B**) PVDF_A, (**C**) PVDF/nHA_R, and (**D**) PVDF/nHA_A, (**E**) PVDF/AU_R (**F**) PVDF/AU_A.

From Figure 2, it is clear that the fibers in all scaffolds exhibit uniform and bead-free morphology. Increasing the collector rotation speed reduces the fiber diameter in all samples. This reduction ranges from an average diameter of 903 ± 54 nm for PVDF_R, 847 ± 70 nm for PVDF/nHA_R, and 890 ± 81 nm for PVDF/AU_R at low collector rotation speed (100 rpm) to values of 347 ± 14 nm for PVDF_A, 290 ± 30 nm for PVDF/nHA_A, and 354 ± 30 nm for PVDF/AU_A at higher collector rotation speed (2000 rpm). Increasing the collector rotation speed leads to the fibers being stretched with greater force along their length. As a result of this stretching, the fibers become thinner, which means that their diameter decreases [31]. High rotation speed leads to increased molecular orientation along the fiber axis (e.g., [32]). SEM images revealed a uniform distribution of nanoparticles in the fibers without visible agglomerations (see Figure 2C–F). The addition of nHA increases the dielectric properties and electrical conductivity of the solution [33]. This causes an increase in repulsive interactions in the solution, which in turn increases the jet pulling force (reduces Rayleigh instabilities) which led to a slight decrease in the fiber diameter (903 ± 54 nm, 847 ± 70 nm for PVDF_R, PVDF/nHA_R and 347 ± 14 nm and 290 ± 30 nm for PVDF_A and PVDF/nHA_A, respectively) (Figure 3) [34].

**Figure 4:**
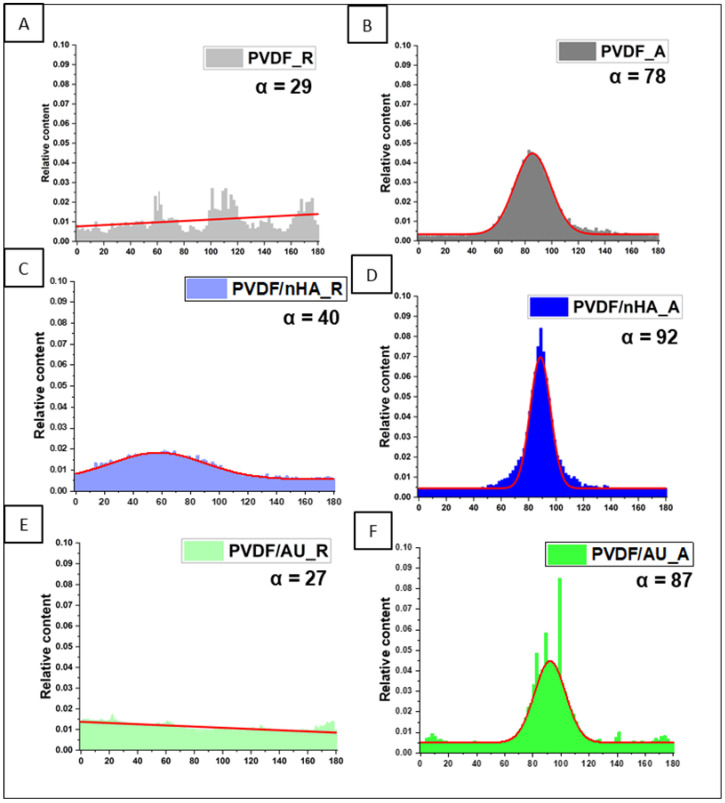
Orientation distributions and anisotropy index α of all nanofibrous scaffolds (**A**) PVDF_R, (**B**) PVDF_A, (**C**) PVDF/nHA_R, and (**D**) PVDF/nHA_A, (**E**) PVDF/AU_R (**F**) PVDF/AU_A.

Figure 4 shows that increasing the collector rotational speed improves the fiber orientation, leading to an orientation with a narrow distribution for fibers collected at 2000 rpm, contrary to randomly arranged fibers collected at 100 rpm. The fiber orientation distribution’s full width at half maximum (FWHM) decreases from 87.90 at 100 rpm to 22.40 at 2000 rpm for pure PVDF. Higher rotational speeds increase tensile forces, enhancing the fibers’ stretching and alignment. The anisotropy index (α) confirms this tendency. As the rotational speed increased from 100 to 2000 rpm, the α value increased from 0.29 to 0.78 for pure PVDF, from 0.40 to 0.92 for PVDF with the addition of nHA, and from 0.27 to 0.87 for PVDF with AuNPs addition. It is worth mentioning that the addition of nHA influenced the slight orientation already in the sample with random fiber orientation, which may be caused by a higher electrical charge in material (PVDF/nHA_R, Figure 4C) [35]. PVDF contains molecular dipoles, and the addition of nHA increases dielectric constant, leading to stronger repulsive interactions in solution, and hence promoting their directionality [36]. In the case of pure PVDF and with AuNPs addition, there is practically no preferred orientation in nonwovens collected at 100 rpm.

The analysis of porosity and average pore size shows the influence of collector rotation speed as well as nHA and AuNPs addition (Table 1). Increasing collector rotation speed leads to lower porosity of nonwovens and slightly lower pore size. Porosity increases slightly with the addition of nHA and AuNPs but is still lower in the aligned fiber orientation. The addition of nHA and AuNPs increases porosity in PVDF/nHA_R and for PVDF/nHA_A (analogously for samples with AuNPs addition), but still the porosity in aligned samples is lower. Thus, the influence of collector rotation speed on fiber porosity can be observed for pure PVDF samples and for samples with nHA and AuNPs additions. The average pore size was reduced with the introduction of a higher collector rotation speed for all samples. The largest difference was observed for samples with nHA addition.

The presence of nHA and AuNPs as additives to PVDF has been verified by SEM-EDS (Table 2). In the case of samples with nHA and AuNPs, analysis confirmed the presence of elements of nHA nanoparticles, such as phosphorus (P) and calcium (Ca), in addition to the basic PVDF elements carbon (C) and oxygen (O). Additionally, the presence of gold was confirmed in both samples PVDF/AU_R and PVDF/AU_A.

### 2.2. Water Uptake, Contact Angle Measurements, and Surface Free Energy Determination

**Figure 5:**
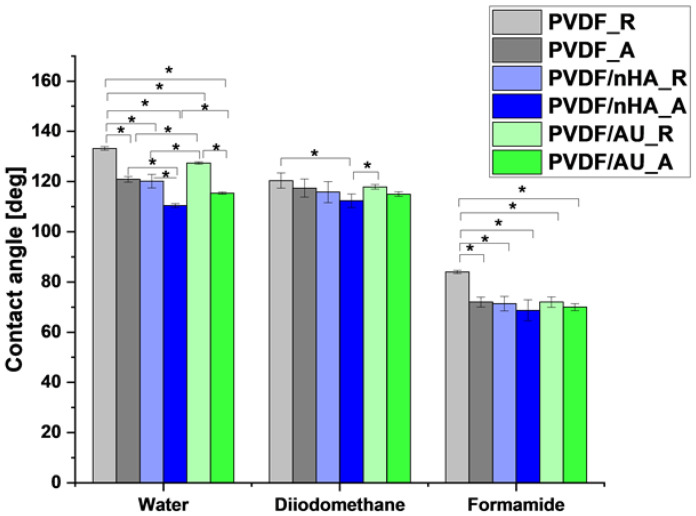
Contact angle measurements for all nanofibrous scaffolds (“*” means *p*-value ≤ 0.05).

Figure 5 illustrates the contact angle measurements, and additionally Table 3 shows the surface free energy values and their components. The overall effect of the addition of nHA, AuNPs, and fiber configuration on the contact angle is visible. The higher collector rotational speed during the electrospinning process, the addition of nHA and AuNPs causes a decrease in the contact angle. A similar phenomenon is observed for aligned fibers arranged compared to randomly oriented fibers. The Figure 5 shows that the contact angles measured with formamide are lower than those obtained with water and diiodomethane. However, the influence of nHA, AuNPs, and fiber configuration is similar for all liquids. The reduction in contact angle in nHA- and AuNPs-doped PVDF samples may also indicate improved cell adhesion to the nanofiber surfaces, which is beneficial in tissue engineering, where it is crucial to ensure adequate cell adhesion to scaffolds to improve tissue regeneration [37].

**Figure 6:**
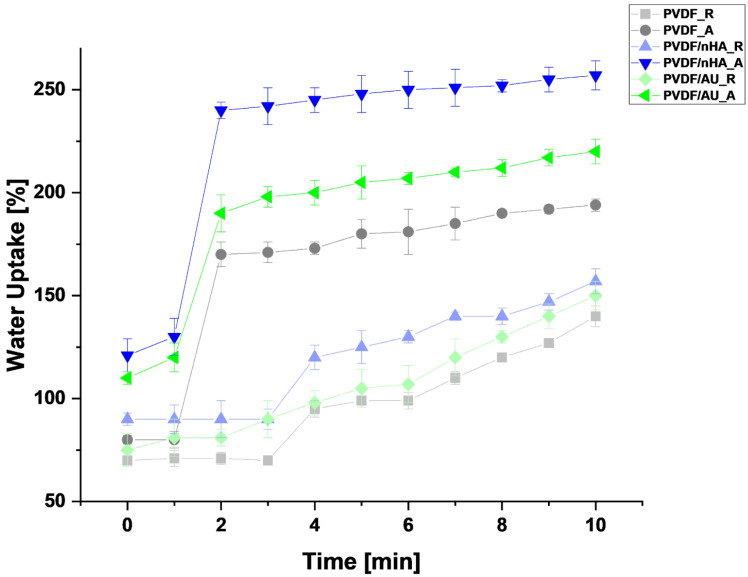
Water uptake of all piezoelectric scaffolds calculated from Equation (8).

It is evident from Figure 6 that the highest water uptake was in scaffolds with nHA in both random and aligned orientation. Pure samples PVDF_R and PVDF_A samples showed lower water absorption in all cases. After 10 min, the water absorption in the PVDF_R sample was about 140%, compared to 194% for PVDF_A. Such low water absorption capacity is consistent with the hydrophobic nature of PVDF, which does not exhibit strong interactions with water. The random arrangement of fibers did not favor increasing the contact surface with water, which additionally limited the ability of the samples to absorb moisture [38]. In the case of oriented PVDF fibers, the higher result can be explained by the more ordered fiber structure, which increases the surface availability for water, resulting in a slightly higher level of absorption. However, the water absorption of pure PVDF still remained low, due to its nonpolar nature [39].

The introduction of nHA and AuNPs increased the ability of the samples to absorb water. The samples with nHA showed significantly higher water absorption capacity. For the PVDF/nHA_R the water absorption was about 157% after 10 min. Hydroxyapatite promoted moisture retention and increased the sample’s ability to absorb water. However, the random arrangement of the fibers limited the full use of this potential, which is why water absorption was lower than in the case of an aligned structure. On the other hand, in the aligned structure, PVDF/nHA_A showed the highest level of water absorption among all the tested samples—about 257% after 10 min. The combination of nHA, AuNPs and the organized structure of the fibers promoted maximization of the contact surface with water, which resulted in significantly better moisture absorption. Such high water absorption capacity could be beneficial in the context of applications in bone/neural tissue engineering, where good moisture retention and stimulation of biological regenerative processes are required [40]. In summary, fiber orientation has a clear effect on the results, with aligned fibers promoting higher water uptake in all samples, suggesting that the topography of the material may be an important factor influencing its hydrophilic properties [41]. The SFE was calculated using the Kaelble-Owens-Wendt method [42]. SFE measurements confirm that the samples with aligned fiber orientation exhibit higher values (see Table 3). More precisely, SFE measured at 35 ± 0.8 mN/m and 45 ± 0.5 mN/m and 38 ± 1.3 mN/m for random PVDF_R and PVDF/nHA_R, and PVDF/AU_R, respectively, compared to 37 ± 0.2 mN/m for aligned PVDF_A, 47 ± 1.1 mN/m for PVDF/nHA_A, and 42.9 ± 1.9 for aligned PVDF/AU_A. The growing order of SFE is PVDF_R > PVDF_A > PVDF/AU_R > PVDF/AU_A > PVDF/nHA_R > PVDFA/nHA_A. In all samples, the dispersive component was higher compared to the polar component and was the main factor influencing the SFE. Moreover, the polar components increased with the addition of nHA and AuNPs, which was more effective in the case of aligned fibers. Thus, the surface polarity (*Xp*) of PVDF/nHA_A was higher and was *Xp* = 0.271 compared to PVDF_A *Xp* = 0.156. Low crystallinity and molecular ordering lead to lower surface polarity [43,44,45], which was observed in the samples with random fiber orientation. The addition of nHA and AuNPs increases the surface polarity in PVDF/nHA_A and PVDF/AU_A samples due to the low polar component $\gamma_{s}^{p}$ of about 8 mN/m and the associated lower WCA [46]. The surface polarity promotes protein adsorption, which has been shown for fibronectin [47]. According to available studies, this ion exchange with the surrounding environment leads to material degradation and the formation of microporosity, which in turn increases the water absorption capacity [48]. Both the material porosity and the fiber porosity resulting from the addition of nHA and AuNPs can have a significant impact on moisture absorption. In summary, the addition of nHA and AuNPs significantly increased the SFE of the samples and their water absorption capacity (see Figure 6), which is crucial for porous biomaterials used in tissue engineering [49,50,51]. 

**Table 3:** SFE, water contact angle (WCA), and their components for all nanofibrous scaffolds.

Sample ID	Water Contact Angle [°]	Dispersive Component [mN/m]	Polar Component [mN/m]	Surface Free Energy [mN/m]	Surface Polarity (*Xp*)	Water Uptake [%]
PVDF_R	133 ± 0.7	32 ± 0.7	3 ± 0.4	35 ± 0.8	0.093	140 ± 5
PVDF_A	120 ± 1.1	32 ± 0.4	5 ± 0.7	37 ± 0.2	0.156	194 ± 3
PVDF/nHA_R	120 ± 2.7	37 ± 0.1	8 ± 0.3	45 ± 0.5	0.216	157 ± 6
PVDF/nHA_A	110 ± 0.7	37 ± 0.6	10 ± 1.2	47 ± 1.1	0.271	257 ± 7
PVDF/AU_R	127 ± 0.5	34 ± 1.2	4 ± 0.6	38 ± 1.3	0.117	150 ± 7
PVDF/AU_A	115 ± 0.4	35 ± 0.9	7± 0.9	42 ± 0.9	0.211	120 ± 6

### 2.3. Determination of the Crystallinity and the Phase Content

#### 2.3.1. Fourier Transform Infrared Spectroscopy (FTIR)

**Figure 7:**
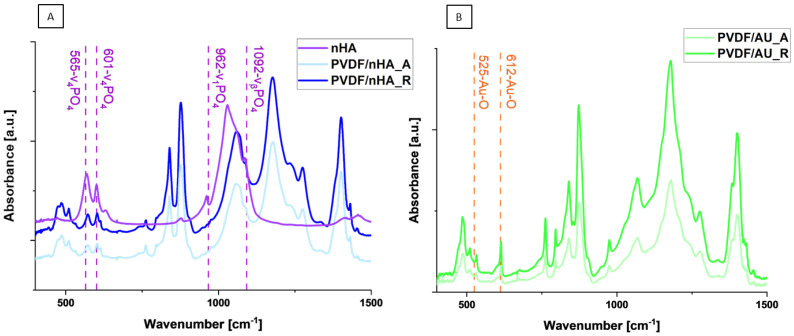
(**A**) FTIR spectra of hydroxyapatite powder and PVDF samples with nHA addition in random and aligned fiber orientation; (**B**) FTIR spectra of AuNPs and PVDF samples with AuNPs addition in random and aligned fiber orientation.

It is evident from Figure 7A,B that both additives nHA and AuNPs were successfully added to PVDF nanofibrous scaffolds. Figure 7A shows the spectra of PVDF samples with nHA added in random and aligned fiber orientation, as well as the measurement of nHA. In PVDF samples, there are three characteristic peaks, 565 cm^−1^ and 601 cm^−1^, corresponding to PO_4_ bending (*ν*_4_) and PO_4_ stretching (*ν*_1_), 962 cm^−1^ corresponding to PO_4_ stretching (*ν*_1_) and 1092 cm^−1^ which can be assigned to PO_4_ bending (*ν_β_*) [52,53]. This confirms the presence of nHA in the structure of PVDF samples. Figure 7B shows the FTIR spectra of PVDF samples with AuNPs. An observed peak at around 525 cm^−1^ and 612 cm^−1^ is relative to the Au-O stretching vibration [54,55,56] and confirms the presence of AuNPs in the nanofibrous scaffolds.

**Figure 8:**
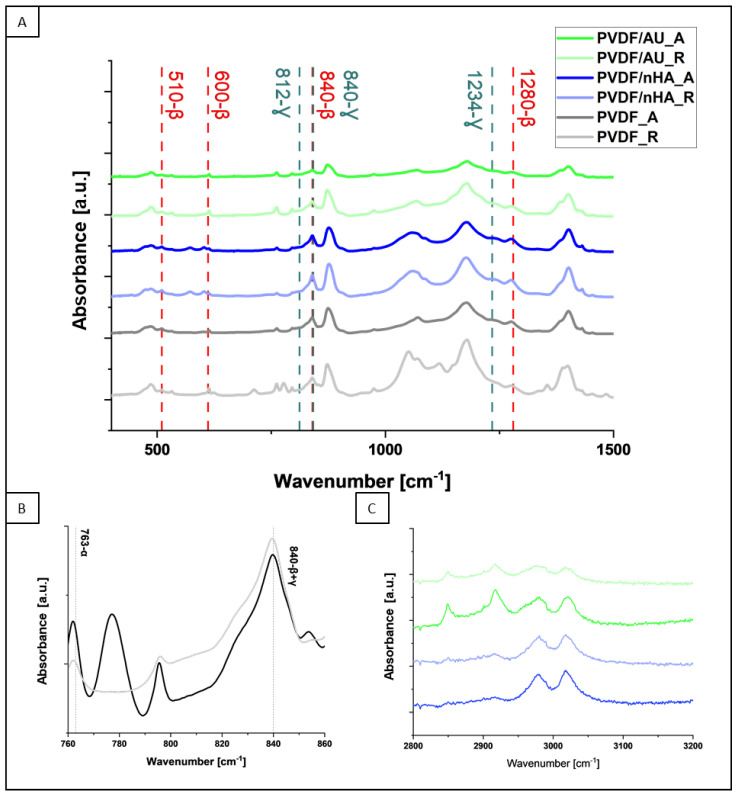
FTIR spectra of PVDF samples with nHA and AuNPs addition in random and aligned samples (**A**) with focus on 760 cm^−1^ to 880 cm^−1^ region (**B**), and 3200 cm^−1^ to 2800 cm^−1^ region (**C**). The graph shows the occurrence of piezoelectric β and γ phases, omitting the bands of non-piezoelectric α phase.

Figure 8 exhibits the FTIR spectra of all samples. The bands from α-phase (490, 766, 1402, 1432 cm^−1^), β-phase (510, 600, 840, 1280 cm^−1^) and γ-phase (812, 840, 1234 cm^−1^) are present [57]. Some of the α and β bands are overlapped with the bands coming from the γ-phase, for instance, with the band at 840 cm^−1^. Multiple phases such as α, β, and γ phases coexist with partially overlapped bands (Figure 8A). Additionally, the peak at 1407 cm^−1^ attributes ν_as_(CC) and _ω_(CH_2_) modes of vibration because of the electronegativity difference of fluorine atoms in PVDF and the surface charge of AuNPs, and nHA. This interaction of PVDF with nHA minimizes the potential energy of stereochemical conformation and induces β, and γ-phases. These particular characteristics have been observed by slight shifts of FTIR spectra corresponding to piezoelectric phases by the addition of nHA to the PVDF matrix [58,59]. Attention was focused on the spectrum in the range of 760 cm^−1^ to 880 cm^−1^, where α- and β-phase peaks are illustrated in detail (Figure 8B) [60]. The differences between the heights of these peaks were used to calculate the piezoelectric phases using Equations (5)–(7). In Figure 8C, peaks of polar β-phases are observed at 3019 and 2980 cm^−1^. These peaks are due to ν_as_(CH_2_) and ν_s_(CH_2_) vibrational bands. AuNPs and nHA cause the minor shifting towards higher wavelength corresponding to lower energy states. This is evidence for the strong interactions of nHA, AuNPs with PVDF [61].

**Figure 9:**
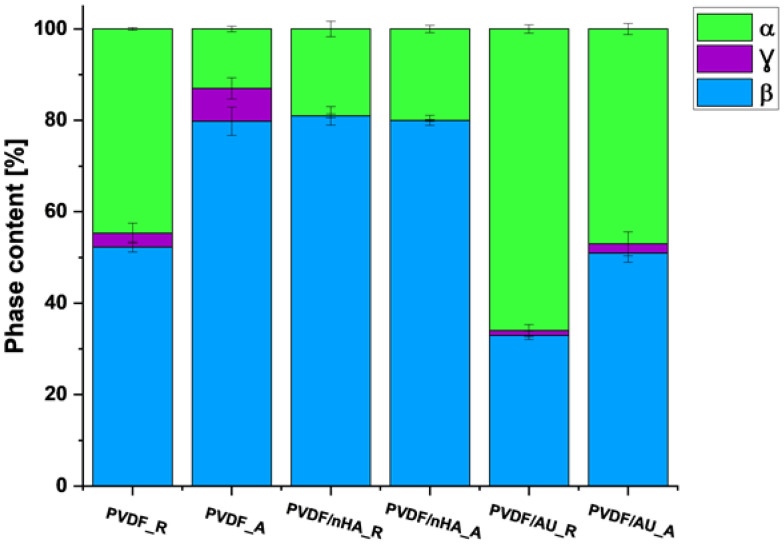
Phase content from FTIR analysis.

The relative amount of β-phase in each sample was estimated from absorption bands at 840 cm^−1^ and 766 cm^−1^ (corresponding to β and α phases, respectively) using Equations (3)–(5) (Table 4, Figure 9). For pure PVDF scaffolds, it is obvious that the β-phase content is higher for the fibers formed at a collector speed of 2000 rpm than for the fibers formed at a collector speed of 100 rpm. Therefore, it can be concluded that the piezoelectric effect will show higher values for the fibers formed at a collector speed of 2000 rpm. The calculated β + γ phase content for the pure PVDF samples is 55.3 ± 2.4 for PVDF_R and 79.8 ± 0.1 for PVDF_A. These calculations confirm the increased amount of β and γ phases in the sample spun at higher collector speeds similar to our previous results [62]. Moreover, it was observed that the addition of nHA increased the amount of piezoelectric phases, in both, random and aligned piezoelectric scaffolds. The strongest effect was exerted by the addition of nHA, with the piezoelectric phase content of 81.9 ± 0.8 for the PVDF/nHA_A sample (including 0.901 ± 1.1 γ-phase), followed by 81 ± 0.5 (including 0.9003 ± 0.4 γ-phase) for the PVDF/nHA_R sample. The addition of nHA can modify the local PVDF matrix, promoting the formation of an ordered chain structure, which favors the occurrence of the β-phase. According to Islam et al. [63] nHA leads to stabilization of β structure, reducing the tendency of its transformation into non-piezoelectric phases. The decrease in the content of piezoelectric phases in PVDF/AU_R (34 ± 1.9) and PVDF/AU_A (53 ± 7.3) may be due to some heating during the sonication, which could lead to a partial transformation of the β and γ phases into the α phase [64]. This issue needs further studies.

**Figure 10:**
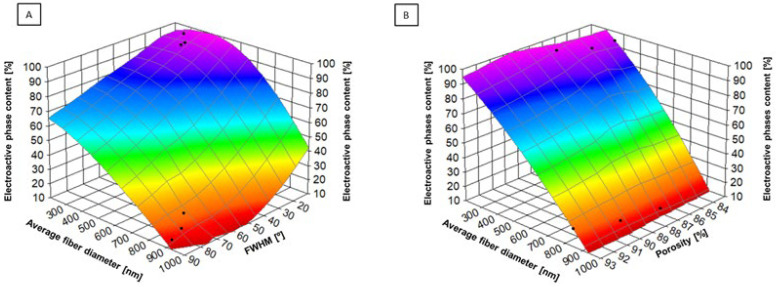
(**A**) The relation between fiber alignment (expressed as FWHM values), average fiber di-ameter, and piezoelectric phases content. (**B**) The relation between average fiber diameter, porosity, and piezoelectric phases con-tent.

Figure 10A demonstrates the relationship between the degree of fiber alignment (expressed as FWHM values), the average fiber diameter, and the piezoelectric phases (β and γ) content. Such a strong increase in the piezoelectric phases content with increasing collector rotation speed, with a simultaneous lower fiber diameter, is associated with an increase in tensile forces, which leads to better alignment and orientation of molecules in nanofibers [65]. It is known from the literature that the orientation of molecules induced by an external field, such as a mechanical one [66], promotes the formation of piezoelectric phases. Similarly, Figure 10B shows that lower porosity in the samples causes an increase in the amount of piezoelectric phases, with a simultaneous lower average fiber diameter. In materials with lower porosity, a higher compactness of the structure promotes the stabilization of the piezoelectric phases, which have higher piezoelectricity compared to the α phase (non-piezoelectric). The β and γ phases are more stable in more ordered and less porous materials because this limits the migration and deformation of the polymer chains [67].

#### 2.3.2. Wide Angle X-Ray Scattering (WAXS)

**Figure 11:**
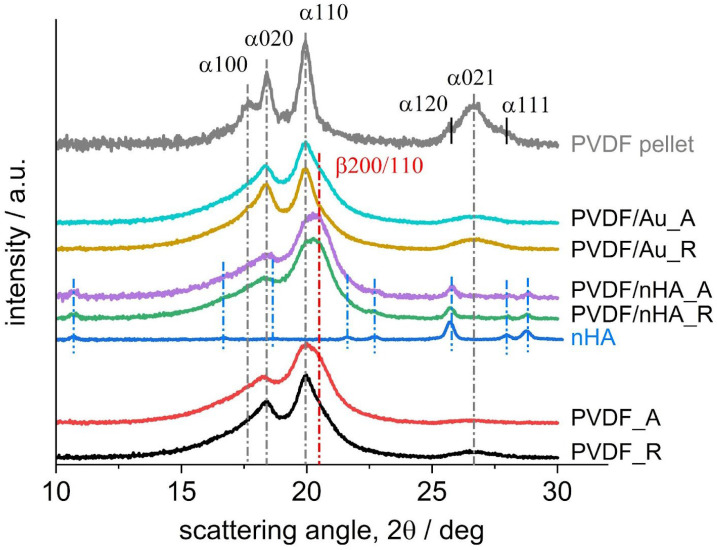
WAXS profiles registered for the scaffolds. Profiles for PVDF pellet melt-crystallized at 10 K/min and for pure nHA, provided for comparison, were used during the peak deconvolution analysis. Profiles registered at room temperature.

Figure 11 illustrates WAXS profiles for all scaffolds. It is supplemented with the profile registered for PVDF pellets crystalized from melt at 10 K/min for comparison. The *hkl* indices of the α and β crystal planes reflections of PVDF at observed diffraction peaks are provided according to the literature [68], and indicated by extended dark grey and red, respectively, vertical dash-dotted lines for guidance: α100, α020, α110 and α021 for the α phase evidenced at c.a. 17.7°, 18.4°, 20° and 26.6° and β200/110 for the β phase evidenced at c.a. 20.8°. Additionally, the profile registered for pure nHA is shown (without *hkl* indices) with indications of the positions of eight diffraction peaks by vertical blue dash-dotted lines. No reflections for Au in PVDF/Au fibers were noticed in the range studied.

**Figure 12:**
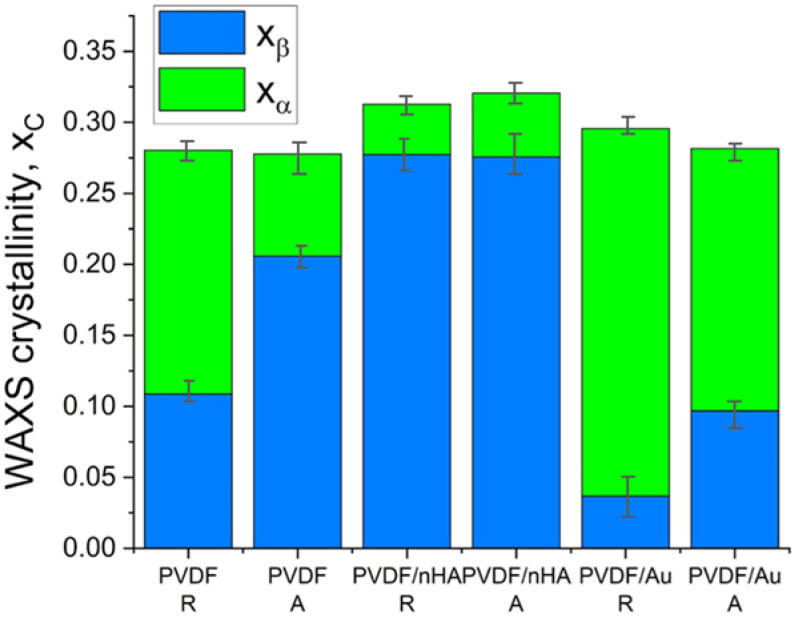
Phases content results obtained from WAXS analysis.

The strongest reflections from the α and β phases: α110 and β200/110 are expected at very similar diffraction angles: 2θ = 20.13° and 20.8°, respectively. Additionally, they are superimposed with a broad amorphous halo, with a maximum expected at c.a. 18–19°. This makes analysis of the profiles registered for the scaffolds quite difficult. However, it is more reasonable to judge the α phase content from the intensity of the diffraction peaks at higher diffraction angles, 2θ > 25°, expected to come from three reflections: the α120, α021 and α111 planes, which are clearly resolved on the profile registered for PVDF pellet crystalized at 10 K/min. The positions of those three diffraction peaks, according to the literature, [68], are expected at 2θ = 25.77°, 26.73° and 27.97°, respectively. On the other hand, the profiles registered for the scaffolds show only one broad diffraction peak and based on its position, this peak was assigned to the reflection from the α021 plane. According to the intensity of the α021 peak, it may be judged from Figure 12 that the highest α phase content may be expected in ultrasonicated PVDF/Au random fibers. Furthermore, decreasing content of α phase may be expected in the order: PVDF/Au aligned > PVDF random > PVDF aligned > PVDF/nHA random and aligned.

In order to determine the scaffolds’ crystallinity and the contents of the α and β phases as well as of nHA, deconvolution of the diffraction peaks was performed by using the non-linear least-square fitting method using Gauss function. It was necessary to start with determination of the interrelations between the intensities of the α phase peaks. For this, the profile registered for PVDF pellet was used, as it shows very high content of the α phase only. Then, according to the data provided in [68], indicating that the structure factors for the α020 reflection are the same for the antipolar and polar forms of the α phase, the α020 peak intensity was taken as the reference. According to our deconvolution results for the PVDF pellet (see Supplementary Materials), the intensity of the strongest α phase reflection, i.e., α110 was assumed as 2.55 times of the α020 intensity. Moreover, according to the literature [68], the structure factor for the α120 reflection in the polar form of α phase was assumed as equal to zero, while the structure factors for the reflections from the α021 and α111 planes for antipolar and polar α phases were assumed equal. In deconvolution analysis of the scaffolds’ profiles, presence of only the α021 reflection was assumed for simplicity, without resolving the α111 reflection.

Figure 12 presents the results of the overall crystallinity and the phase content for the scaffolds. The overall crystallinity was determined as c.a. 30% (see Supplementary Materials for details). It may be seen that addition of nHA strongly increases the β phase content in PVDF from 10 to 27% in random fibers and from 20 to 27% in aligned fibers. On the other hand, ultrasonication leads to increase in the α phase content, from 17 to 25% in the PVDF/Au random fibers and from 7 to 17% in the PVDF/Au aligned fibers. It may also be concluded that higher rotation speed of the collector (aligned fibers) increases the β phase content in PVDF and PVDF/Au fibers but does make no change in PVDF/nHA fibers.

As regards the α and β phase content, the WAXS results generally agree with the FTIR results. It has to be noted that in the diffraction angle range applied in the WAXS methodology, we could not reveal the presence of the γ phase. The indication of the presence of the γ phase was found by DSC method, what is reported in next section.

#### 2.3.3. Differential Scanning Calorimetry (DSC)

**Figure 13:**
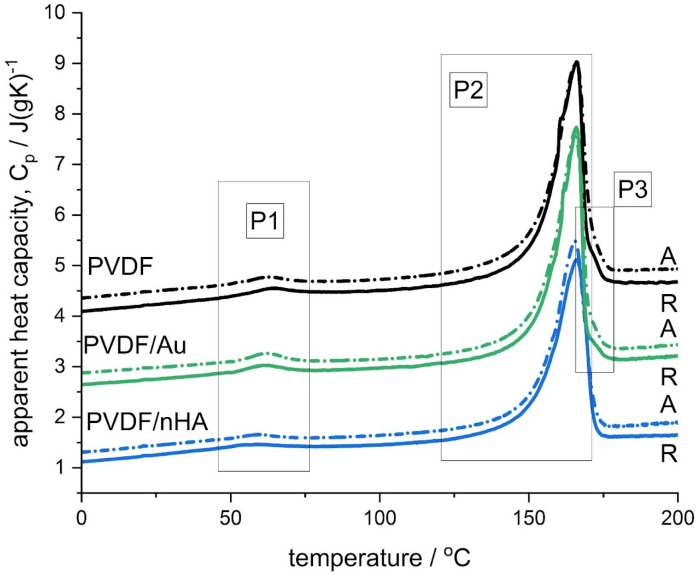
DSC scans registered during the 1st heating shown as the apparent heat capacity. The curves were shifted for clarity.

Figure 13 shows the DSC scans registered during 1st heating for all the samples. On the curves characteristic features are marked: two endothermic peaks with apparent maxima and one shoulder denoted as P1, P2, and P3, respectively, where the peak P1 appears around 60 °C, the peak P2 around 166 °C and the shoulder P3 around 171 °C. It has to be noted that for samples doped with nHA the peak P2 occurs without the shoulder P3.

The peak P1, which is usually detected in PVDF samples after storing at ambient conditions, is attributed to disruption of very defective secondary crystals. The peak temperature range and size was found comparable to the results reported for PVDF samples casted from DMF solution at various temperatures [69] and to the results from the studies on the effect of storage and annealing in PVDF [70].

**Figure 14:**
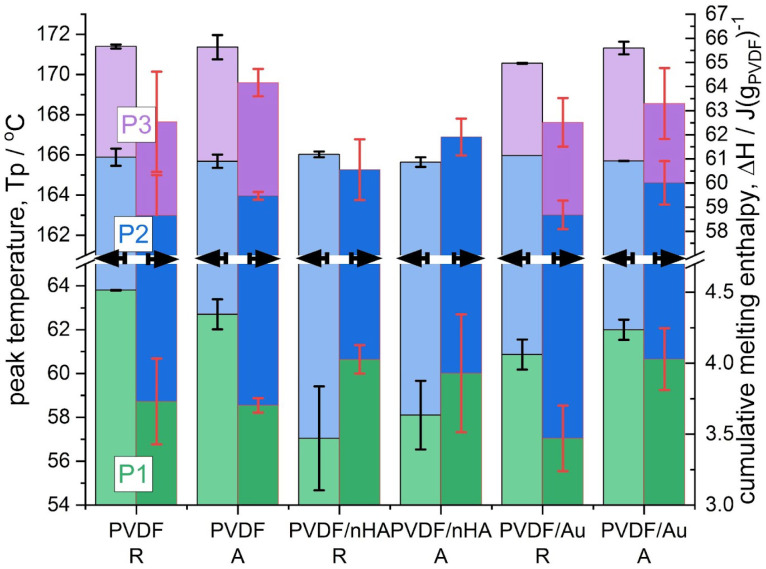
Characteristics of the peaks P1, P2 and P3 determined after deconvolution of the DSC scans (see Figure 13): maximum of the peak temperature, *Tp*, (left axis), and the cumulative melting enthalpies normalized to the PVDF content, Δ*H* (J/g_PVDF_) (right axis).

As regards the high-temperature melting thermal effect consisting of the peaks P2 and P3, observed only in PVDF and PVDF/Au samples, these thermal effects were subjected to a peak deconvolution analysis using Asymetric Double Sigmoid (ADS) function, which resulted in determination of the contribution of each peak (P2 and P3). The temperature position and the melting enthalpy, Δ*H*, of each peak (P1, P2, P3) are presented in Figure 14. Moreover, the deconvolution analysis examples are provided in the Supplementary Materials, also for PVDF/nHA samples, where approximation of the melting thermal effect was done with a single ADS function.

Figure 14 shows that the temperature position of the P1 peak, which is attributed to disruption of very defective secondary crystals, depends on the fiber composition or post-processing (sonication). Pure PVDF fibers show the highest temperature positions of the P1 peak, c.a. 64 and 63 °C, for random and aligned fibers, respectively. The lowest temperature positions of the P1 peak are seen for PVDF/nHA fibers, c.a. 57 and 58 °C, for R and A fibers, respectively. For sonicated PVDF/Au fibers, the positions of the P1 peak were found lower than for pure PVDF, at temperatures c.a. 61 and 62 °C, for R and A fibers, respectively.

The temperature position of the P2 peak is seen at c.a. 166 °C for all the samples, while of the P3 peak c.a. 171 °C, observed for PVDF and PVDF/Au fibers only. As revealed by our FTIR and WAXS analysis (see the previous Section 2.3.2) showing always the presence of the α and β phases, the P2 peak was assigned to the simultaneous melting of the α and β crystals. On the other hand, the P3 peak was assigned to the melting of the γ crystals as revealed by our FTIR analysis, which is also supported by data in the literature [71].

For PVDF/nHA fibers the size of the peak P1 was found slightly higher than for pure PVDF, c.a. 4 J/gPVDF, along with the highest size of the peak P2, 57 and 58 J/gPVDF for random and aligned fibers, respectively.

Ultrasonication of PVDF/Au fibers led to slight decrease in the size of the P1 peak for random fibers, from 3.7 to 3.4 J/gPVDF, and to slight increase in the size of the P1 peak for aligned fibers, from 3.7 to 4 J/gPVDF. The size of the P2 was found to increase for random fibers without a change in the size of the P3 peak. On the other hand, for aligned fibers the size of the P3 peak decreased without a change in the size of the P2 peak. It may be concluded that ultrasonication of aligned fibers led to a slight decrease in γ phase while increasing the amount of very defective secondary crystals, indicated by increase in the size of the P1 peak. For random fibers, ultrasonication led to a slight increase in the α crystals from the very defective secondary crystals, manifested by a decrease in the size of the P1 peak. Both conclusions are supported by our FTIR results (see Section 2.3.1, Table 4 and Figure 9).

Assuming the theoretical value of the equilibrium fusion enthalpy, Δ*H*^0^ = 104.5 J/g [72], crystallinities for the scaffolds were determined as c.a. 60%, which is two-fold higher than the values obtained by WAXS analysis (c.a. 30% in Figure 13). Such a huge discrepancy is highly unexpected and the presumed difference should not exceed a few percent in favor of DSC values. This has been confirmed for PVDF pellet showing 53.5% WAXS and 55.8% DSC crystallinity (see Supplementary Materials). Similarly small differences in crystallinity as determined by WAXS and DSC for various PVDF samples can be found in the literature [73]. Much larger difference between DSC and WAXS crystallinity is usually observed in fibers formed by electrospinning technique (e.g., [74]). Such a difference can be easily explained by the highly defective crystal structure of electrospun fibers, formed during extremely fast solvent evaporation. This structure with relatively low crystallinity is registered by WAXS experiment, contrary to the DSC analysis where crystallinity is deduced from melting of crystals formed both during electrospinning as well as during heating. Taking into account that the structure of electrospun fibers is very far from the thermodynamic equilibrium, the process of additional crystallization/recrystallization is naturally expected during DSC heating, resulting in apparently high crystallinity. The process of growing of small and imperfect crystals during heating is unresolved in the heat flow since it is stretched out on a relatively long time (temperature range). According to the literature [75], for polymer fibers there exists the effect of the crystal melting point depression with decreasing fiber diameter below the bulk melting point. Contrary to this, we found slightly higher melting temperatures, *Tp*, for the fibers than for the pellet (165 °C), which might be another indication of the crystals’ growing/perfecting upon heating.

Additional reason of higher melting enthalpies could be related to the fact of using the same value of the equilibrium melting enthalpy (Δ*H*^0^ = 104.5 J/g [72]), irrespectively of the phase content. Assumption of this value for α phase only, and a much higher value, for example *ΔH*^0^*_β_* = 219.7 J/g [69] for the β phase, could provide better, however, still limited approximation of the DSC and WAXS results. In such an approach, good approximation was obtained only for the β phase-rich PVDF/nHA fibers but not for the samples with low content of the β phase (pure PVDF and PVDF/Au fibers).

### 2.4. In Vitro Study on Osteoblasts Human MG-63 Cell Line

**Figure 15:**
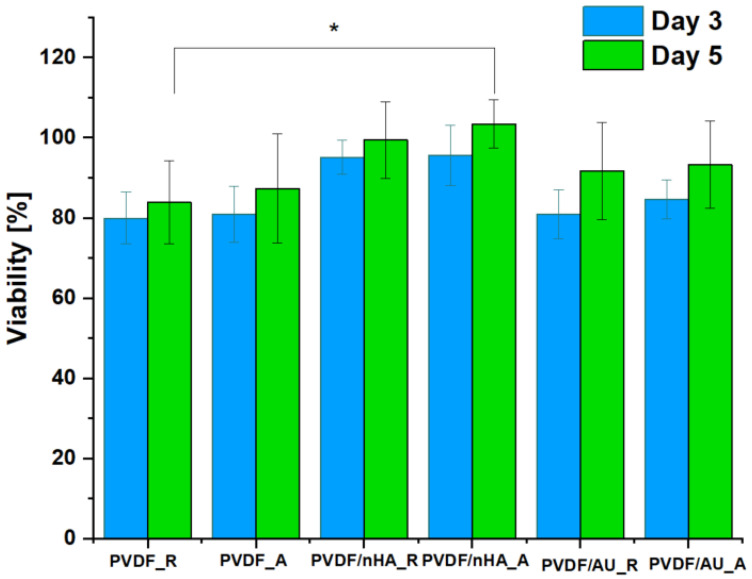
Cell viability of MG-63 cells cultured on pristine and with nHA and AuNPs addition PVDF nanofibrous scaffolds as the rate of TCP (Tissue Culture Plastic, 100%). Statistical significance: * *p* < 0.05.

Osteoblasts are crucial for ongoing bone remodeling and play a significant role in assessing materials used in bone tissue engineering [76]. In addition to evaluating cellular viability, we investigated the interaction between osteoblasts and nonwoven scaffolds. Cell viability was measured for MG-63 cells cultured on pure PVDF scaffolds, as well as those with nHA and AuNPs additives, with both random and aligned fiber orientations (Figure 15). The in vitro analysis confirmed the non-toxic nature of all PVDF nanofibrous scaffolds, with all samples achieving cell viability levels ≥ 70%, meeting the ISO 10993-5 standard [77] for non-toxic materials.

**Figure 16:**
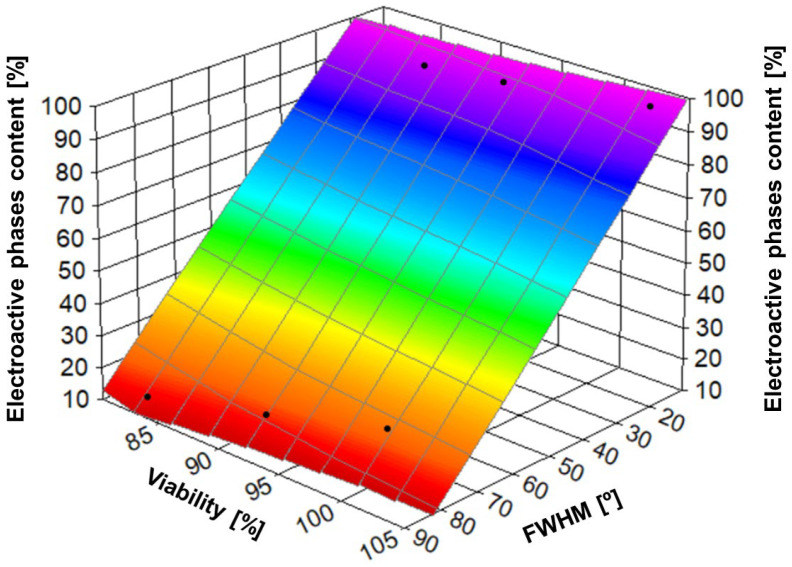
The relation between porosity, piezoelectric phase content, and MG-63 viability after day 5.

Experiments on piezoelectric scaffolds made of pure PVDF, PVDF/nHA and PVDF/AU showed interesting relationships between nanofiber orientation, piezoelectric phase content (mainly β phase) and MG-63 cell viability after 5 days of cell culture (Figure 16). The influence of fiber alignment (expressed as FWHM), and piezoelectric phase content on MG-63 cell viability was significant. The highest MG-63 cell viability was observed in the the sample with nHA addition and aligned fiber orientation (lowest FWHM), which were additionally characterized by the highest β phase content. Hydroxyapatite, due to its bioactivity, promoted better bone cell deposition and growth [78]. Moreover, aligned PVDF fibers with high β phase content additionally enhanced biological processes, promoting piezoelectric activity, which in turn stimulated MG-63 cells. The alignment of the fiber with the cell growth direction promoted more organized proliferation and better cell attachment to the scaffold surface. On the contrary, on PVDF_R samples, MG-63 cells showed moderate viability, which can be attributed to the lower β-phase content and less favorable structure for cell adhesion and proliferation. The random fiber orientation limits the mechanical and electrical stimuli that affect the cells. The addition of AuNPs promoted an increase in the piezoelectric phase. AuNPs supported the stabilization of the β-phase through surface interactions, which contributed to the increased piezoelectric phase content. Samples with AuNPs showed better results in terms of cell viability compared to pure PVDF, regardless of the fiber orientation. Both AuNPs and nHA could act as an additional factor supporting cell adhesion and proliferation by improving the electrical conductivity and supporting the piezoelectric properties of PVDF. In the case of aligned samples, cell growth was even more noticeable, indicating the synergistic effect of fiber orientation and additives. 

In summary, there is a relationships between nanofiber orientation (expressed as FWHM), piezoelectric phase content, and MG-63 cell viability (Figure 16). Oriented fibers (lower FWHM) favor higher β-phase content, which in turn improves MG-63 cell viability and proliferation. Modification of PVDF with gold or hydroxyapatite further enhances these effects, making such materials more suitable for bone tissue engineering applications. However, this issue is very complex and requires more detailed research. Analysis of these relationships allows for a better understanding of the influence of the structure and surface modification of materials on their bioactivity and application potential in bone tissue engineering [79].

**Figure 17:**
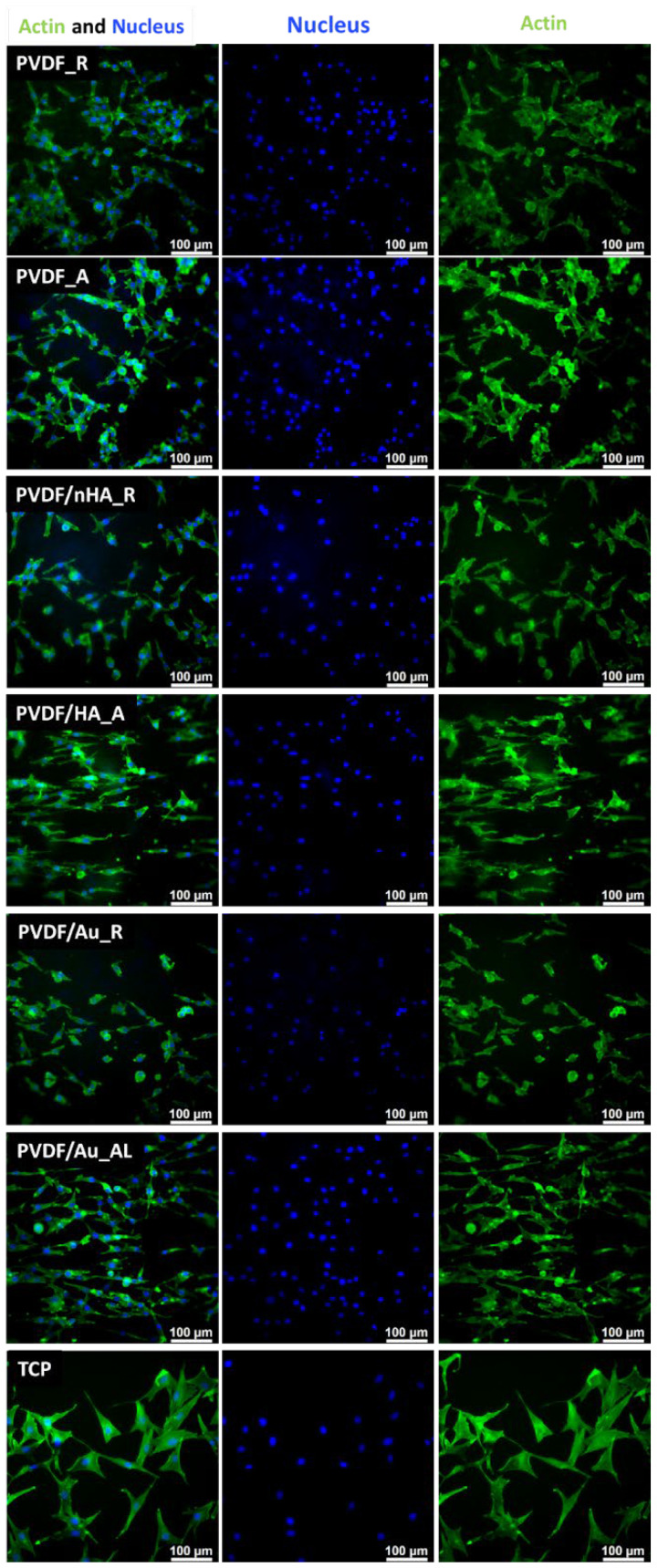
FM images of stained MG-63 directly cultured on the specimen substrate for 3 days. Samples PVDF_R, PVDF_A, PVDF/nHA_R, PVDF/nHA_A, PVDF/AU_R and PVDF/AU_A in comparison to TCP.

**Figure 18:**
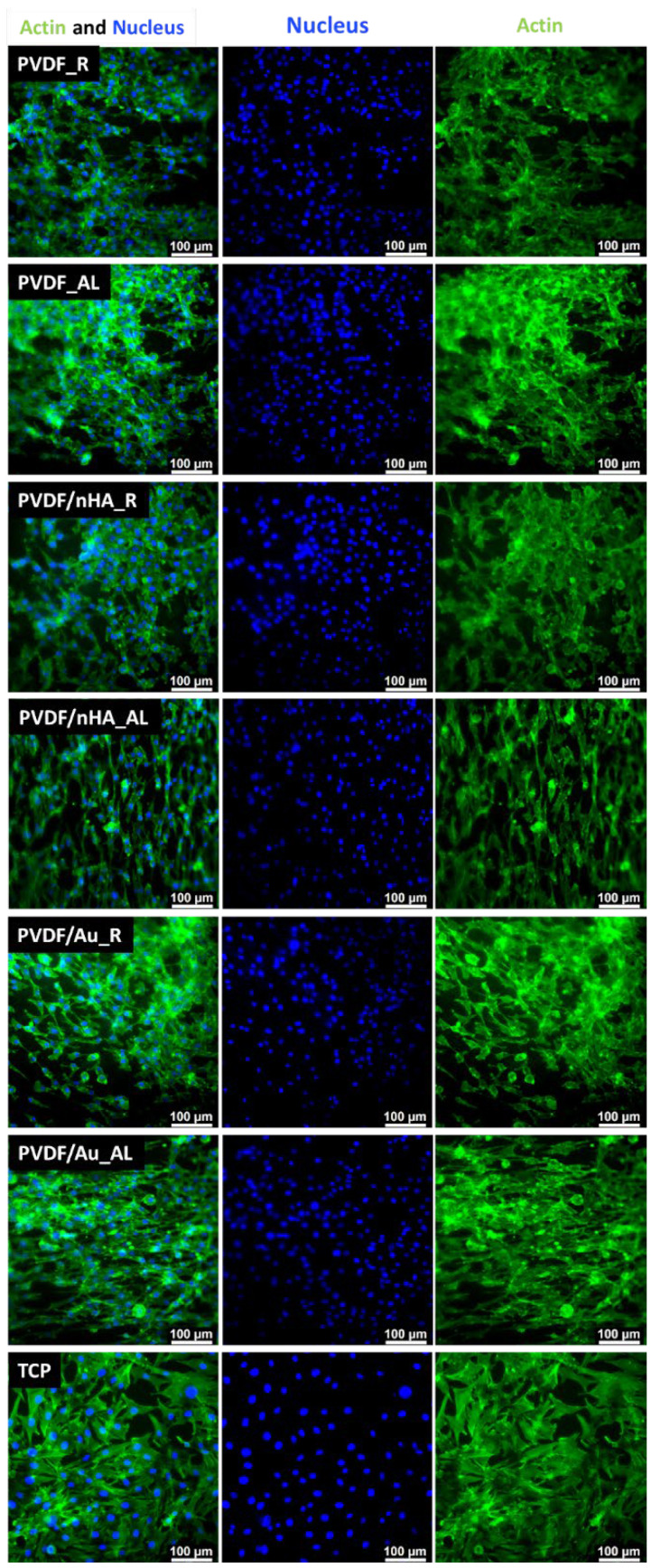
FM images of stained MG-63 directly cultured on the specimen substrate for 5 days. Samples PVDF_R, PVDF_A, PVDF/nHA_R, PVDF/nHA_A, PVDF/AU_R and PVDF/AU_A in comparison to TCP.

Cell proliferation and attachment are critical for tissue regeneration, representing the early stages of the process [80]. To further evaluate this, cellular morphology was assessed. After 3 and 5 days of culture on pure PVDF scaffolds, PVDF/nHA, and PVDF/AuNPs, immunostained cells were analyzed using fluorescence microscopy (Figure 17 and Figure 18). The morphology and spreading of osteoblasts on the scaffold surfaces closely resembled those on tissue culture plastic (TCP), corresponding with good cell viability. The cells exhibited a well-spread morphology and strong intercellular connections. Effective interaction between osteoblasts and the nonwoven scaffolds was confirmed by the elongation and spreading of the cells, which was especially pronounced on PVDF_A, PVDF/nHA_A and PVDF/Au_A composite nanofibers. Fluorescence microscopy images clearly show different cell growth patterns depending on the nanofiber orientation-cell elongation due to contact guidance is seen on aligned nonwovens. The cells tended to elongate in the direction of the fiber orientation, suggesting that PVDF nanofibers affect the direction of osteoblast migration and proliferation. This behavior may be due to the mechanical interaction of the piezoelectric properties of PVDF, which can generate electric charges, thus promoting cell elongation along the fibers. These effect was observed previously on nonvowens formed from PCL [81], PMMA [44] etc. In the case of scaffolds with a random nanofiber structure, osteoblast cells showed a more dispersed morphology, which is characteristic of an irregularly arranged surface.

Previous research has highlighted the importance of incorporating nanoscale components like nHA and AuNPs into scaffolds to enhance cell proliferation [82,83]. The improved adhesion and proliferation observed on PVDF/nHA and PVDF/Au fibers are likely due to the inclusion of nHA and AuNPs, which contribute to reduced porosity (Table 1) and hydrophobicity (Table 3). In vitro studies support the conclusion that PVDF/nHA and PVDF/Au nanofibrous scaffolds demonstrate increased bioactivity and hold significant potential for promoting bone formation.

### 2.5. In Vitro Study on Human Induced Pluripotent Stem Cell-Derived Neural Stem Cell Culture (hiPSC-NSC)

**Figure 19:**
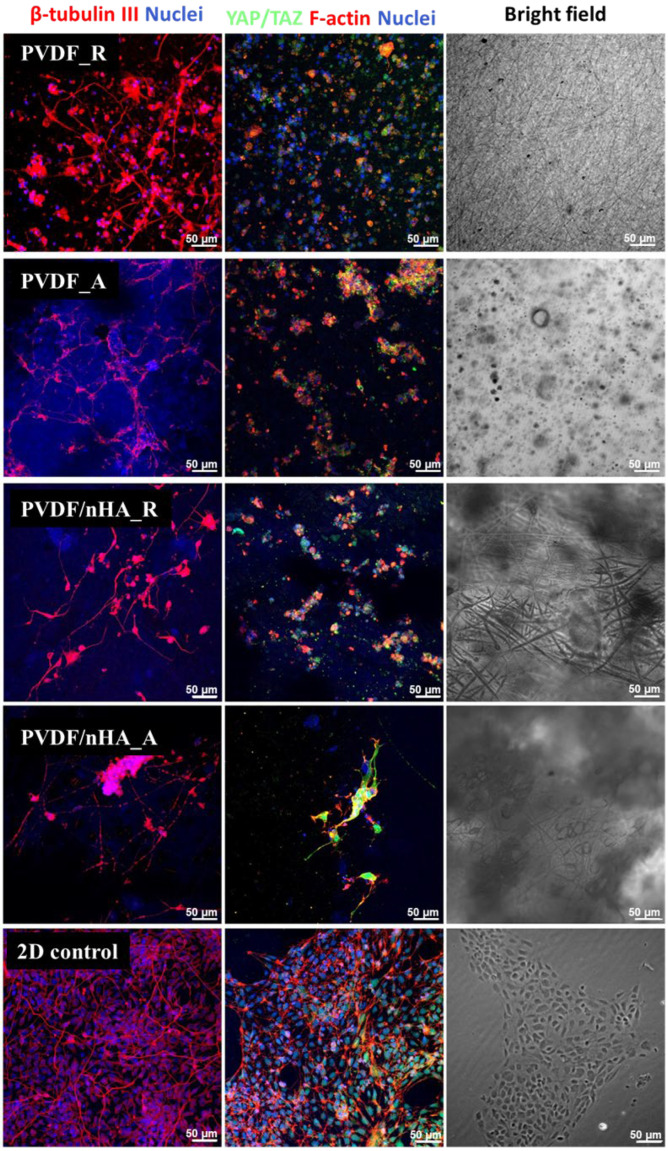
Immunocytochemical analysis of hiPSC-derived NSC cultured for 5 days on the PVDF scaffolds. After 5 days of culture, cells were stained for a neuronal marker (β-tubulin III, red) or mechanotransduction mediating factors YAP/TAZ (green) together with f-actin staining (Alexa546-conjugated Phalloidin, red). Extracellular matrix-coated (diluted geltrex) glass coverslips served as 2D control. Cell nuclei are contra-stained with Hoechst (blue).

In vitro preliminary experiments using Human Induced Pluripotent Stem Cell-Derived Neural Stem Cell (hiPSC-NSC), were conducted to evaluate cell differentiation and behavior on electrospun nanofibrous mats made from pure PVDF and PVDF doped with nHA, with fibers oriented in both random and aligned configurations (Figure 19). These nanofibers served as scaffolds, supporting the growth of iPSC-derived neural stem cells. YAP/TAZ staining used to visualize mechanosensitive molecules that shuttle between the nucleus and cytoplasm in response to environmental conditions was mainly observed throughout the cells, similar to what is typically seen on soft hydrogels or 3D scaffolds. Cells at the surface of the nanofibers differentiated into neurons, displaying long, interconnected projections (as indicated by beta-tubulin III staining). A slight effect of fiber orientation on cell alignment was observed. This may be attributable to an additional extracellular matrix (ECM) protein coating applied to the nanofibrous mats, which is necessary for adequate cell adhesion. In aligned samples, higher directional differentiation of neuronal cells can be observed compared to random orientation. However, whether aligned fibers better support the formation of neuronal networks and axonal growth compared to random scaffolds requires more detailed studies.

## 3. Experimental Part

### 3.1. Materials and Methods

Poly(vinylidene fluoride) (PVDF) with a molecular weight of Mw = 530,000 g/mol was purchased from Sigma-Aldrich, Steinheim, Germany. Hydroxyapatite (nHA) nanopowder, with particles smaller than 200 nm, was also obtained from Sigma-Aldrich, Germany, as well as dimethylformamide (DMF) and acetone. A solution of citrate-stabilized gold nanoparticles (AuNPs, 40 nm in size, 99.99% purity, optical density of 8.7) was obtained from Nano Flow, Liège, Belgium. Reagents used were of analytical grade.

Reagents for in vitro tests on osteoblasts, such as amino acids, L-glutamine, fetal bovine serum (FBS), and antibiotics (penicillin/streptomycin), were purchased from Sigma Aldrich (Gillingham, UK). Phosphate-buffered saline (PBS), Presto Blue, Trypsin EDTA, Dulbecco’s Modified Eagle Medium, ActinGreen, and NucBlue were purchased from Thermo Fisher Scientific (Waltham, MA, USA). The human osteoblast cell line MG-63 was also purchased from Sigma-Aldrich (86051601, London, UK).

Reagents for in vitro tests on hiPSC-NSC (Thermo Fisher Scientific, USA), such as, Essential 8 Medium, Vitronectin (VTN-N) Recombinant Human Protein, Truncated, UltraPure™ 0.5 M EDTA, pH 8.0, Geltrex™ LDEV-Free hESC-qualified Reduced Growth Factor Basement Membrane Matrix, Neurobasal™ Medium, Gibco™ PSC Neural Induction Medium, Advanced DMEM, Goat serum, Alexa Fluor™ 546 Phalloidin, Alexa Fluor™ 546 goat anti mouse IgG2aAlexa Fluor™ 488 goat anti rabbit H + L were purchased from Thermo Fisher Scientific (USA). Dulbecco’s Phosphate Buffered Saline, Modified, without calcium chloride and magnesium chloride, Accutase^®^ solution, PFA, Triton X-100, Anti-β-Tubulin III (neuronal) antibody, Hoechst 33258, Mouse monoclonal were purchased from Sigma-Aldrich (UK). Rabbit polyclonal anti YAP/TAZ antibody was purchased from Cell Signaling Technology, Danvers, MA, USA, and Fluorescent Mounting Medium was purchased from Dako, Santa Clara, CA, USA.

### 3.2. Preparation of PVDF/nHA Solution and Scaffold Fabrication via Electrospinning Technique and Sonochemical Coating

PVDF pellets were dissolved in DMF/acetone (4:1 ratio) at 60 °C for 6 h. For pure PVDF 20 wt.% of PVDF solutions were prepared. For samples with nHA addition, an appropriate amount of nHA nanoparticles was added to obtain 10 (*w*/*v*%) solution and stirrer for 2 h. To avoid nHA aggregation, the solutions were sonificated for 30 min (ultrasonic cleaner EMAG, EMMI-D60, Salach, Germany).

All the PVDF solutions were loaded into a 5 mL syringe and placed into a Bioinicia horizontal setup (Fluidnatek LE-50, Bioinicia, Valencia, Spain). Based on previous studies [62,84], the process parameters leading to the formation of the higher amount of piezoelectric phases in samples were selected. A 23 G needle was used with an inner diameter of 0.337 mm. A flow rate during process was 0.8 mL/h. The distance between the collector and the needle was 190 mm. The fibers were collected on a rotating drum collector rotating at 100 (to obtain random fibers), and 2000 rpm (to obtain aligned fibers). The process was carried out at the temperature range of 21 °C and humidity of 25%. The positive voltage applied to the needle was 22 kV, and the collector electric potential was −2 kV. After electrospinning, all materials were placed under a fume hood for 48 h in order to evaporate the solvent. The pure PVDF samples were marked as PVDF_R for random and PVDF_A for aligned fiber orientation of the sample. Also, samples with addition of nHA were marked PVDF/nHA_R for random fiber orientation and PVDF/nHA_A for aligned fiber orientation in the sample. For samples with AuNPs, the sonochemical coating was used according previously developed method [85]. The ultrasonic treatment of pure PVDF samples with gold (AuNPs) nanoparticles was carried out in a citric acid bath containing the Au component. Ultrasonic fiber functionalization was achieved with an efficiency of 80% using a pulse cycle on a Hielscher UP200Ht device (Teltow, Germany). The coating process took place at specific time intervals (2 × 2 min). Then, the samples were thoroughly rinsed with water and dried. These samples were marked as PVDF/Au_R for random and PVDF/Au_A for aligned fiber orientation with AuNPs addition.

### 3.3. Characterization of Piezoelectric Nanofibrous Composites

#### 3.3.1. Morphology Analysis

The morphological analysis of piezoelectric scaffolds was performed using Scanning Electron Microscopy imaging (SEM, JSM-6010PLUS/LV InTouchScope™, JEOL, Tokyo, Japan), operated at an accelerating voltage of 11 kV. Before imaging, each nonwoven sample was double-coated with a thin layer of gold (2–3 nm). After imaging, analysis of microstructures was conducted using ImageJ software (1.52q software version, Washington, DC, USA). Fiber diameter distribution was determined by applying a Gaussian approximation, with 100 measurements taken for each sample.

The alignment of the fibers analyzed using ImageJ software with the directionality plugin was quantified by calculating the full-width at half maximum (FWHM) of the Gaussian function used to approximate the orientation distribution. FWHM values were averaged across six images for each sample.

Analysis of the fiber orientation in all specimens was determined using the anisotropy index α, ranging between 1 for the ideal alignment of fibers and 0 for the random distribution of fibers (Equations (1) and (2)) [86,87].

(1) $$\Omega =\frac{1}{I_{tot}}\sum I_{i}\left|\begin{array}{cc} {cos}^{2}\theta_{i} & {sin\theta }_{i}{cos\theta }_{i}\\ sin\theta_{i}cos\theta_{i} & {sin}^{2}\theta_{i} \end{array}\right|$$

(2) $$\alpha =1-\lambda_{1}/\lambda_{2}$$

More precisely, *Ω* is a matrix with *λ*_1_ and *λ*_2_ values, *I_tot_*—the sum of individual nanofiber lengths (nm), *I_i_*—the length of the nanofiber (nm), *θ_i_*—the angle between the *x*-axis and the axis of the nanofiber oriented perpendicular to the collector axis.

Porosity (*p*) was determined from Equation (3) [88]:

(3) $$p=\frac{V_{t}-V_{f}}{V_{t}}=\left(1-V_{f}\times \frac{\rho }{m_{t}}\right)=(1-\frac{m_{f}}{m_{t}})$$

where, *V_f_* is the volume of the tested sample, mf is the mass of the tested sample, m_t_ is the theoretical mass of the solid sample as the product of the PVDF density (1.75 g/cm^3^ [89]) and the volume *V_t_*.

Pore size (*P*) was estimated from Equation (4) [88]:

(4) $$P=\frac{2D}{\left(1-p\right)}$$

where, *D* is the mean fibers diameter in each specimen, (1 − *p*) is the area of fibers per unit and *p* is the porosity.

The presence of nHA and AuNPs was confirmed through Energy Dispersive X-ray Spectroscopy (EDS) (JSM-6010PLUS/LV InTouchScope™, JEOL, Tokyo, Japan). Samples were measured with the following parameters: working distance (WD) = 10 mm, accelerating voltage of 8 kV, probe current of 500 pA, and a collecting time of 30 min.

#### 3.3.2. Water Uptake, Water Contact Angle Measurements and Surface Free Energy Determination

Piezoelectric samples was divided into a 2 × 1 cm rectangle and immersed in demineralized water every 60 s for 10 min and weighed (XA 52.R2 analytical balance, Radwag, Radom, Poland).

The water uptake capacity of the samples was determined using Equation (5) [90].

(5) $$\%\;Water\;uptake=100\times \frac{W_{wet}-W_{o}}{W_{o}}$$

where, *W_o_* and *W_wet_* is the weight of dry and wet samples, respectively.

The wettability of nanofibrous scaffolds was tested using the Data Physics OCA 15EC contact angle goniometer (Filderstadt, Germany). A drop of liquid (volume 2 µL) was deposited on the surface of the nanofibrous scaffolds. The contact angle was calculated after 3 s at 21 °C.

Surface Free Energy (SFE) was calculated using the Kaelble-Owens-Wendt method. This method assumes that SFE is the sum of two main independent components related to polar and dispersion interactions. Three liquids were used to evaluate SFE-water, highly dispersible diiodomethane, and formamide, as described [87] (Equations (6) and (7)).

(6) $${{(\gamma }_{s}^{d})}^{0.5}=\frac{\gamma_{d}\left(cos\theta_{d}+1\right)-\sqrt{\left(\frac{\gamma_{d}^{p}}{\gamma_{w}^{p}}\right)}\gamma_{w}(cos\theta_{w}+1)}{2(\sqrt{\gamma_{d}^{d}}-\sqrt{\gamma_{d}^{p}-(\frac{\gamma_{w}^{p}}{\gamma_{w}^{p}})})}$$

(7) $${{(\gamma }_{s}^{p})}^{0.5}=\frac{\gamma_{w}\left(cos\theta_{w}+1\right)-2\sqrt{\gamma_{s}^{d}\gamma_{w}^{d}}}{2\sqrt{\gamma_{w}^{p}}}$$

where, $\gamma_{s}^{d}$—dispersion component of SFE, $\gamma_{s}^{p}$—polar component, $\gamma_{d}$—SFE of diiodomethane; $\gamma_{d}^{d}$—dispersive component of diiodomethane, $\gamma_{d}^{p}$—polar element of diiodomethane, $\gamma_{w}$—SFE of water; $\gamma_{w}^{d}$—the dispersive element of water, $\gamma_{w}^{p}$—polar element of water; $\theta_{d}$ and $\theta_{w}$ contact angle of diiodomethane and water, respectively.

Surface polarity (*Xp*) was determined using the Wu’s method (Equation (8)) [91]:

(8) $$X_{p}=\frac{\gamma_{s}^{p}}{\gamma_{s}}$$

#### 3.3.3. Determination of the Crystallinity and the Phase Content

##### Fourier Transform Infrared Spectroscopy (FTIR)

Molecular structure analysis, determination of piezoelectric phase content and presence of nHA and AuNPs was conducted using Fourier Transform Infrared Spectroscopy with ATR technique (Bruker Vertex 70, Mannheim, Germany). The results are data from measurements of five samples. Investigations of the nanofibrous scaffolds was provided in the range of 400 cm^−1^ to 4000 cm^−1^ with a resolution of 2 cm^−1^ and a total of 32 scans.

The amount of piezoelectric phases (*F_EA_*), β and γ, was calculated from Equation (9) [36,92]:

(9) $$F_{EA}=\frac{I_{840*}}{\left(\frac{K_{840*}}{K_{763}}\right)\times I_{763}+I_{840*}}\times 100\%$$

where, intensities from bands 763 cm^−1^ and 837–841 cm^−1^ were calculated. According to the Beer-Lamber law, absorption coefficients K_840_ and K_763_ are 7.7 × 10^4^ cm^2^ mol^−1^ and 6.1 × 10, respectively. Absorbance from bands 1234 cm^−1^ and 1275 cm^−1^ was used to calculate the *β*-phase (F(*β*)) and *γ*-phase (F(*γ*)) [36,92].

(10) $$F\left(\beta \right)=F_{EA}\times \left(\frac{\Delta H_{\beta^{\prime }}}{\Delta H_{\beta^{\prime }}+\Delta H_{\gamma^{\prime }}}\right)\times 100\%$$

(11) $$F\left(\gamma \right)=F_{EA}\times \left(\frac{\Delta H_{\gamma^{\prime }}}{\Delta H_{\beta^{\prime }}+\Delta H_{\gamma^{\prime }}}\right)\times 100\%$$

where Δ*Hβ′* is the absorbance difference between the peaks (1275 cm^−1^ and 1260 cm^−1^), whereas Δ*Hγ′* is the peak 1234 cm^−1^ and the nearest valley, around 1225 cm^−1^.

##### Wide Angle X-Ray Scattering (WAXS)

Bruker D8 Discover diffractometer (Manheim, Germany) was used. Coupled theta-2theta geometry with divergent beam optic (1 mm slit) was used in reflective mode with Cu lamp X-ray source (power was set to nominal 1600 W). Scans were done at 10–30 deg. 2theta angular range, with 1 s signal record time per step (0.02°. increment step size).

The WAXS profiles were analysed using the non-linear least-square fitting method with the Gauss function resulting to deconvolution of the diffraction peaks, enabling determination of the overall crystallinity, *X_C_*, and the α and β phase contents, *X_α_* and *X_β_* as:

(12) $$X_{c}=\frac{\sum I_{i}}{I_{tot}},\;X_{\alpha }=\frac{\sum I_{i\alpha }}{I_{tot}},\;X_{\beta }=\frac{\sum I_{i\beta }}{I_{tot}}$$

where *I_i_* represents integral intensity of an *i*-th diffraction peak, *I_i__α_* and *I_i__β_* are the intensities of the *α* or *β* phase diffraction peaks, respectively, and *I_tot_* represents the total integral intensity of the registered WAXS profile.

##### Differential Scanning Calorimetry (DSC)

Thermal analysis of PVDF scaffolds was performed using a Differential Scanning Calorimeter (DSC, Pyris 1, Perkin Elmer, Waltham, MA, USA) equipped with Intracooler 2P under nitrogen atmosphere. Oriented and random fibrous samples (7–10 mg) as well as a pellet sample for comparison were loaded into standard aluminum pans.

Thermal analysis consisted of determination of the melting temperatures and enthalpies as well as calculation of the crystallinity after a deconvolution analysis of the melting endotherms using Asymmetric Double Sigmoid (ADS) function.

#### 3.3.4. In Vitro Study on Human Osteoblasts MG-63 Cell Line

In vitro research was evaluated using the human MG-63 cell line. The cells were grown in 25 cm^2^ flasks containing medium made from 10% fetal bovine serum (FBS), High-Glucose Dulbecco’s Modified Eagle’s Medium (DMEM), 1% glutamine and antibiotics. Incubation was carried out at 37 °C in an atmosphere containing 5% CO_2_. To detach the cells, the flask was rinsed with PBS, followed by the addition of 3 mL of a 0.05% trypsin solution. The flask was then returned to the incubator for a few minutes. After cell detachment, 10 mL of fresh culture medium was added, and the suspension was centrifuged at room temperature. The resulting pellet was re-suspended in culture medium to obtain the desired cell density. Several experiments were performed to evaluate the cellular response to monolithic nanofibers, including cytotoxicity tests using extracts and analysis of cellular morphology.

To evaluate cellular viability, MG-63 cells were seeded onto the test samples and Tissue Culture Plate (TCP) at a density of 1 × 10^4^ cells per well, followed by incubation. On days 3 and 5, the culture medium was carefully removed, and each well was filled with 180 µL of PBS and 20 µL of Presto Blue reagent. The plate was then incubated for 60 min. Fluorescence readings were obtained using excitation/emission filters of 530/620 nm with a Fluorescent Accent FL device from Thermo Fisher Scientific. The results were analyzed by comparing the Presto Blue fluorescence of blank samples, which served as a baseline for no metabolic activity, and the control (TCP), which represented 100% cell viability.

Fluorescence microscopy was used to examine the morphology of osteoblasts in direct contact with the material. MG-63 cells were cultured on electrospun membranes, and after 3 and 6 days of incubation, the cells were fixed using 3% formaldehyde for 20 min. To permeabilize the cell membranes, the fixed cells were treated with 0.01% Triton X-100 for 5 min. Subsequently, the cellular nuclei and cytoskeleton were stained for 30 min using a solution containing ActinGreen and NucBlue, which specifically bind to the cytoskeleton and nuclear DNA, respectively. Microscopic images were acquired using a Leica AM TIRF MC microscope (Wetzlar, Germany).

#### 3.3.5. In Vitro Study on Human Induced Pluripotent Stem Cell-Derived Neural Stem Cell Culture (hiPSC-NSC)

Neural stem cells were derived from a human induced pluripotent stem cell (hiPSC) line following the protocol described by Buzanska et al. [93], with some modifications. Briefly, cells from a human episomal iPSC line (Gibco, Thermo Fisher Scientific) were routinely cultured on vitronectin-coated culture dishes in Essential 8 Medium (Thermo Fisher Scientific). For neural induction, the cells were washed in PBS without calcium and magnesium (PBS−/−) and passaged using EDTA. The cells were then transferred to Geltrex-coated (Thermo Fisher Scientific) culture vessels in Essential 8 Medium. After 24 h, the medium was replaced with PSC Neural Induction Medium (Thermo Fisher Scientific), and the cells were cultured for up to 7 days, with daily medium changes. On day 7 of neural induction, the confluent cultures were washed with PBS−/− and passaged using Accutase into new Geltrex-coated (1:100 in Neurobasal Medium) 6-well plates at a density of 1 × 10^5^ cells/cm^2^ in neural expansion medium (a 1:1 mixture of PSC Neural Induction Medium and Advanced DMEM, Thermo Fisher Scientific). The cells were maintained under these conditions and passaged weekly. Cells from passage 6 were used for further experiments.

##### Cell Culture on Biomaterials and Immunofluorescent Staining

To culture hiPSC-NSCs on biomaterials, PVDF scaffolds were sterilized by exposure to UV light for 30 min on each side, then rinsed with 70% ethanol, washed three times with PBS for 10 min each, and incubated with a Geltrex solution (1:100 in Neurobasal Medium) for 1 h at 37 °C. The hiPSC-NSCs were seeded onto the PVDF scaffolds at a density of 1 × 10^5^ cells/cm^2^ in neural expansion medium. An uncoated scaffold was used to assess the adhesion of the neural stem cells directly to the PVDF biomaterial. The next day, the scaffolds were transferred to a new culture dish and cultured for up to 7 days. On day 7, the cells on the PVDF scaffolds were washed with PBS, fixed with 4% paraformaldehyde (PFA), washed again with PBS, and permeabilized with 0.5% Triton X-100 for 15 min. After removing the permeabilization solution, the samples were blocked with 10% goat serum for 1 h at room temperature. The cells were then incubated overnight at 4 °C with the following primary antibodies: monoclonal mouse anti-neuronal beta III tubulin (1:1000, Sigma-Aldrich), polyclonal rabbit anti-YAP/TAZ (1:500, Stem Cell Signaling), and Alexa-546-conjugated Phalloidin (1:500, Thermo Fisher Scientific). The next day, after washing with PBS, the samples were incubated for 1 h at room temperature with the appropriate secondary antibodies: Alexa-546 goat anti-mouse IgG2a and Alexa-488 goat anti-rabbit polyclonal antibody (both 1:500, Thermo Fisher Scientific). After washing with PBS, the samples were counterstained with Hoechst (1:100, Sigma) for 15 min to visualize the nuclei. Following a final PBS wash, the samples were mounted in fluorescent mounting medium (Dako, Glostrup, Denmark) and imaged using a confocal microscope (LSM510, Zeiss, Oberkochen, Germany) at the Laboratory of Advanced Microscopy Techniques, Mossakowski Medical Research Institute, PAS.

### 3.4. Statistical Analysis

All results were analyzed to determine statistical significance. Viability results were evaluated using GraphPad Prism 8.0.1 software, applying a threshold of *p* < 0.05. As needed, a two-way ANOVA followed by Tukey’s multiple comparisons test was performed. Statistical significance was assessed based on *p*-values, with values below 0.05 considered significant. *p*-values in the range of 0.01 to 0.05 were marked with an asterisk (“*”) to indicate significance.

## 4. Conclusions

It was presented that higher collector rotational speed or nHA addition to PVDF electrospun fibers increases not only the fibers’ alignement but also promotes formation of the piezoelectric phases. The β phase content was found the highest in PVDF/nHA fibers, being 80–90% of the total crystallinity and was found independent on the collector rotational speed. The lowest β phase content was determined for ultrasonicated PVDF/Au random fibers, being 12–33% of the total crystallinity. For pure PVDF and ultrasonicated PVDF/Au fibers, higher β phase content was observed for higher collector rotational speed. Additionally, low content of the γ phase in pure and with AuNPs addition, being 3–7% of the total crystallinity, was found in FTIR analysis and confirmed using DSC analysis as a small high temperature melting peak. Moreover, addition of nHA or coating of the fibers with AuNPs improved scaffolds’ surface properties (SFE) and water absorption.

According to WAXS analysis, the fibers were c.a. 30% crystalline, only, which is much lower than the crystallinity of a pellet (c.a. 53%). During DSC heating, high metastability of electrospun structure led to intense growth of the defective small crystals resulting in twice higher melting enthalpy and slightly higher melting temperature than for the pellet samples.

In vitro results using MG-63 results showed that aligned fibers favor higher β-phase content, which in turn improves MG-63 cell viability and proliferation. Modification of PVDF with nHA or AuNPs further enhances these effects, making such materials more suitable for bone tissue engineering applications [94]. Whereas, in vitro results using hiPSC-NSC showed that in aligned samples, higher directional differentiation of neuronal cells can be observed. This is due to that aligned fibers better support the formation of neuronal networks and axonal growth compared to random scaffolds [95].

The piezoelectric composites demonstrated a high degree of adaptability in their fiber orientation (random vs. aligned), offering flexibility in their applications. Randomly oriented fibers may better mimic the structural complexity of a natural bone matrix, while aligned fibers are particularly suited for guiding nerve regeneration, highlighting the material’s multifunctionality.

The conducted studies on piezoelectric nanofibrous composites with nHA and AuNPs confirmed the value of these innovative composites as promising candidates for advancing tissue engineering solutions, particularly in challenging regenerative contexts like bone and nervous system repair.

## Figures and Tables

**Table 1:** Porosity and pore size values for all nanofibrous scaffolds.

	PVDF_R	PVDF_A	PVDF/nHA_R	PVDF/nHA_A	PVDF/AU_R	PVDF/AU_A
Porosity [%]	87 ± 1.1	83 ± 0.9	93 ± 1.1	87 ± 0.6	90 ± 0.4	85 ± 0.3
Pore size [nm]	202 ± 7	195 ± 3	204 ± 9	198 ± 8	203 ± 6	200 ± 4

**Table 2:** Piezoelectric scaffold composition in wt% as measured using SEM-EDS.

Element	PVDF_R	PVDF_A	PVDF/nHA_R	PVDF/nHA_A	PVDF/AU_R	PVDF_AU_A
C	61.81 ± 0.7	61.97 ± 0.6	60.47 ± 0.3	60.13 ± 0.4	60.75 ± 0.5	60.84 ± 0.7
O	38.19 ± 0.3	38.03 ± 0.4	33.9 ± 0.7	33.81 ± 0.6	38.9 ± 0.3	38.83 ± 0.4
Ca	-	-	3.52 ± 0.4	3.79 ± 0.3	-	-
P	-	-	2.11 ± 0.2	2.27 ± 0.1	-	-
Au	-	-	-	-	0.35 ± 0.5	0.33 ± 0.2
Other	-	-	-	-	-	-

**Table 4:** Phase content in nanofibrous scaffolds from FTIR.

Sample ID	F(α) (%)	F(β) + F(γ) (%)	F(β) (%)	F(γ) (%)
PVDF_R	44.7 ± 0.3	55.3 ± 2.4	52.3 ± 1.1	3 ± 2.2
PVDF_A	13 ± 0.6	88 ± 0.1	80.8 ± 3.1	7.2 ± 2.3
PVDF/nHA_R	19 ± 1.7	81 ± 0.5	80.997 ± 2.2	0.003 ± 0.4
PVDF/nHA_A	18.1 ± 0.8	81.9 ± 0.8	80.999 ± 0.2	0.901 ± 1.1
PVDF/AU_R	66 ± 0.9	34 ± 1.9	32.97 ± 0.9	1.03 ± 1.3
PVDF/AU_A	47 ± 1.2	53 ± 7.3	50.96 ± 2	2.04 ± 2.6

## Data Availability

Raw data will be made available by the corresponding author upon reasonable request.

## Supplementary Materials

The following supporting information can be downloaded at: https://www.mdpi.com/article/10.3390/molecules30051041/s1, Figure S1: DSC deconvolution; Figure S2: WAXS deconvolution.
