# Supplementary material for: On the Structural and Biological Effects of Hydroxyapatite and Gold Nano-Scale Particles in Poly(Vinylidene Fluoride) Smart Scaffolds for Bone and Neural Tissue Engineering
Source: Molecules. 2025 Feb 25;30(5):1041. doi: 10.3390/molecules30051041 (PMC11901919; doi:10.3390/molecules30051041)
Supplement: Supplementary file 1 [file molecules-30-01041-s001.zip › molecules-3434015-supplementary.pdf]

molecules

MDPI

Supplementary data

On the Structural and Biological Effects of Hydroxyapatite and Gold Nano-Scale Particles in Poly(Vinylidene Fluoride) Smart Scaffolds for Bone and Neural Tissue Engineering

Angelika Zaszczyńska [1], Marzena Zychowicz [2], Dorota Kołbuk [1], Piotr Denis [1], Arkadiusz Gradys [1,*] and Paweł Ł. Sajkiewicz [1,*]

[1] Institute of Fundamental Technological Research, Polish Academy of Sciences, Pawinskiego 5B, 02-106 Warsaw, Poland

[2] Department of Stem Cell Bioengineering, Mossakowski Medical Research Institute, Polish Academy of Sciences, Pawinskiego 5, 02-106 Warsaw, Poland

* Correspondence: argrad@ippt.pan.pl (A.G.); psajk@ippt.pan.pl (P.Ł.S.)

Figure S1. DSC deconvolution:

Peak Analysis

Data Set:[Book1]PVDF_R_1!M"Cp"		Date:2024-10-14
BaseLine:Constant		
Chi^2=2.07919E-03	Adj. R-Square=9.98227E-01	# of Data Points=2884
SS=5.97352E+00	Degrees of Freedom=2873	

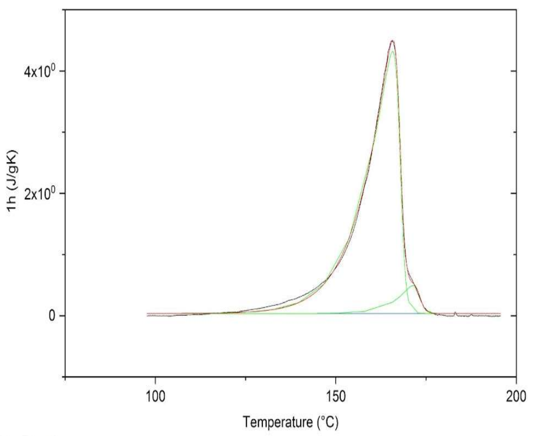

Fitting Results

Peak Index	Peak Type	Center Max	Area Intg	FWHM	Max Height	Area IntgP
1	Asym2Sig	165.72196	51.09264	9.83945	4.28319	93.18134
2	Asym2Sig	171.39612	3.73877	6.87888	0.45692	6.81866

Peak Analysis

Data Set:[Book4]PVDF_A_1!M"Cp"		Date:2024-10-14
BaseLine:Constant		
Chi^2=3.96633E-03	Adj. R-Square=9.96570E-01	# of Data Points=2903
SS=1.14706E+01	Degrees of Freedom=2892	

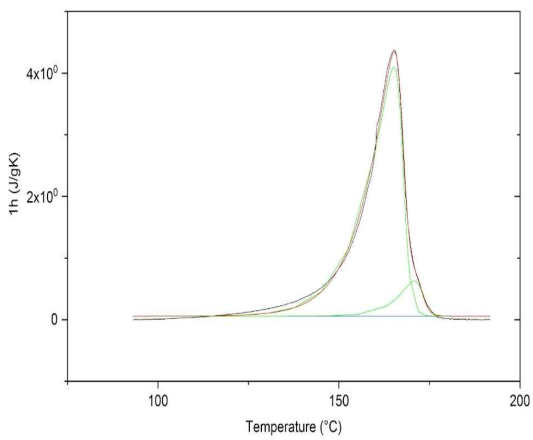

Fitting Results

Peak Index	Peak Type	Center Max	Area Intg	FWHM	Max Height	Area IntgP
1	Asym2Sig	165.15583	51.21556	10.54119	4.03871	90.8507
2	Asym2Sig	170.84205	5.15776	7.75496	0.57259	9.1493

Peak Analysis

Data Set:[Book15]PVDF_AU_R_1!M"Cp" Date:2024-10-14
BaseLine:Constant
Chi^2=3.08277E-03 Adj. R-Square=9.97211E-01 # of Data Points=2872
SS=8.81981E+00 Degrees of Freedom=2861

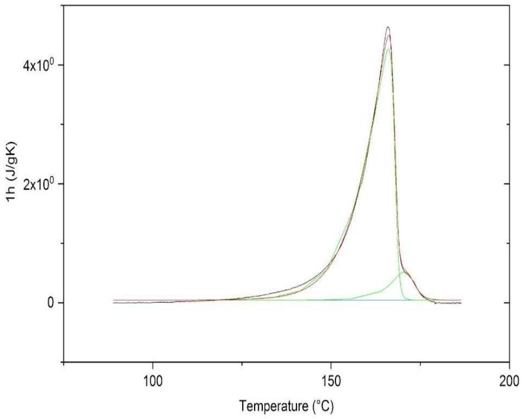

Fitting Results

Peak Index	Peak Type	Center Max	Area Intg	FWHM	Max Height	Area IntgP
1	Asym2Sig	166.08219	46.31377	9.18932	4.22116	91.5881
2	Asym2Sig	170.58687	4.25368	7.75418	0.47243	8.4119

Peak Analysis

Data Set:[Book18]PVDF_AU_A_1!N"Cp" Date:2024-10-14
BaseLine:Constant
Chi^2=1.82058E-03 Adj. R-Square=9.98470E-01 # of Data Points=2422
SS=4.38942E+00 Degrees of Freedom=2411

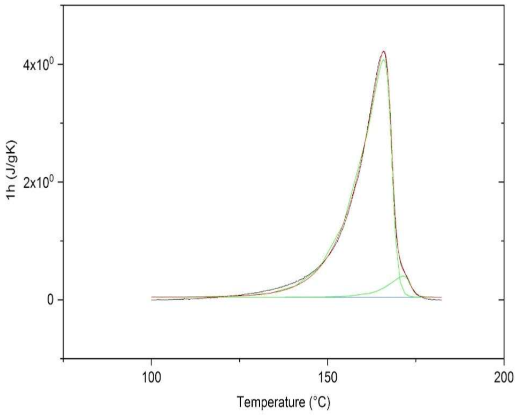

Fitting Results

Peak Index	Peak Type	Center Max	Area Intg	FWHM	Max Height	Area IntgP
1	Asym2Sig	165.84457	48.94172	10.04727	4.02778	94.27006
2	Asym2Sig	171.41221	2.97479	7.21132	0.35571	5.72994

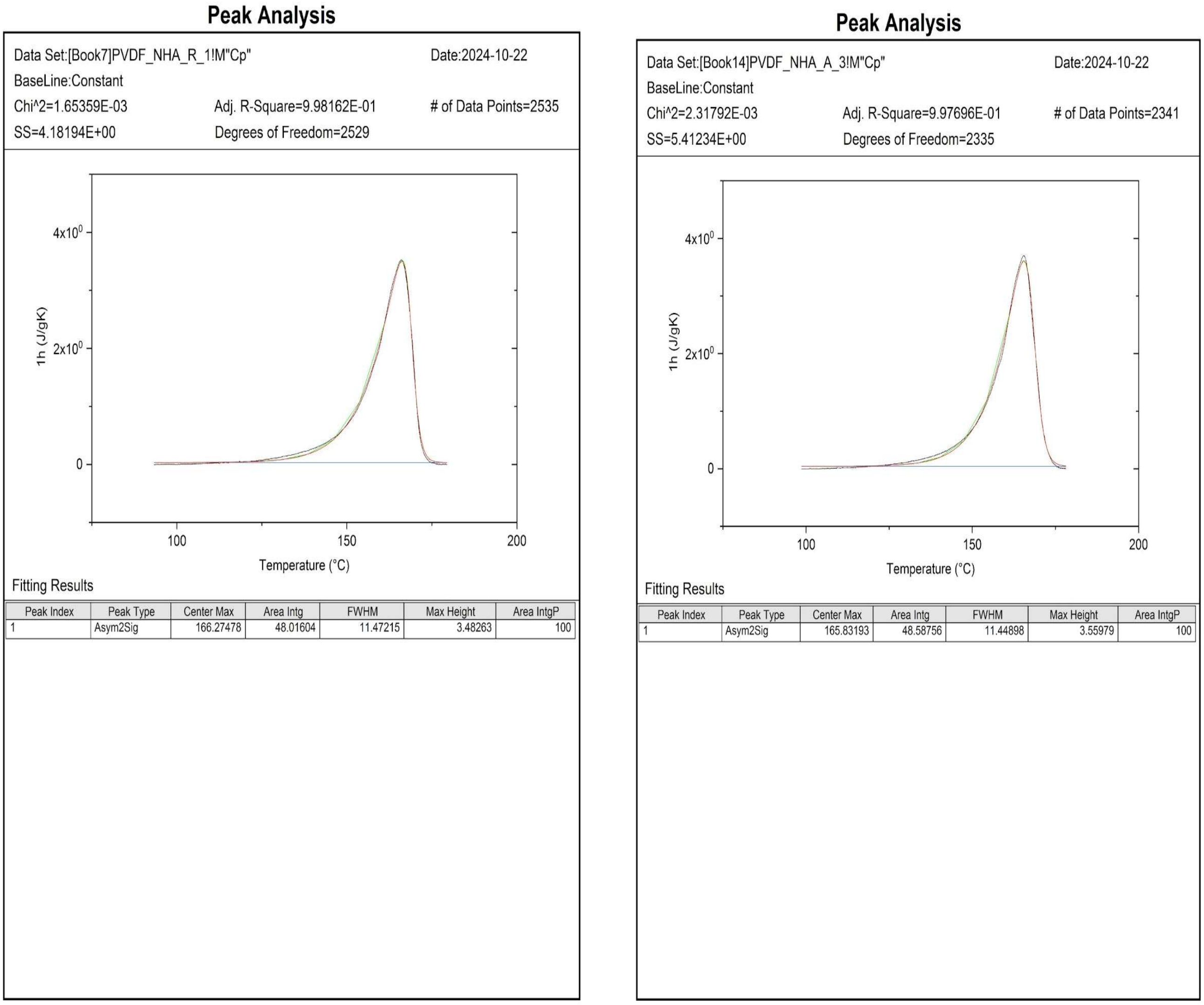

Figure S2. WAXS deconvolution:

Peak Analysis

Data Set:[Book1]IntegResult1!K"PVDF_R" Date:2024-09-19
BaseLine:Straight
Chi^2=8.56520E-06 Adj. R-Square=9.98267E-01 # of Data Points=978
SS=8.20546E-03 Degrees of Freedom=958

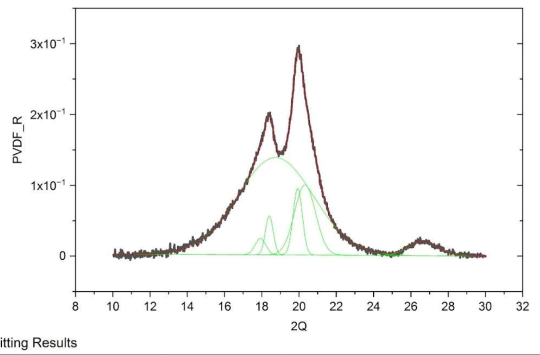

Fitting Results

Peak Index	Peak Type	Area Intg	FWHM	Max Height	Center Grvty	Area IntgP
1	Gauss	0.01637	0.66903	0.02299	17.92681	1.66053
2	Gauss	0.02881	0.49281	0.05492	18.40192	2.92253
3	Gauss	0.05811	0.58129	0.09392	19.92636	5.89488
4	Gauss	0.04109	1.8811	0.02052	26.68882	4.16786
5	Gauss	0.14085	1.33449	0.09916	20.31782	14.28811
6	Gauss	0.70057	4.79276	0.13732	18.7426	71.06609

Peak Analysis

Data Set:[Book1]IntegResult1!L"PVDF_A" Date:2024-09-19
BaseLine:Straight
Chi^2=5.64834E-06 Adj. R-Square=9.98958E-01 # of Data Points=978
SS=5.41111E-03 Degrees of Freedom=958

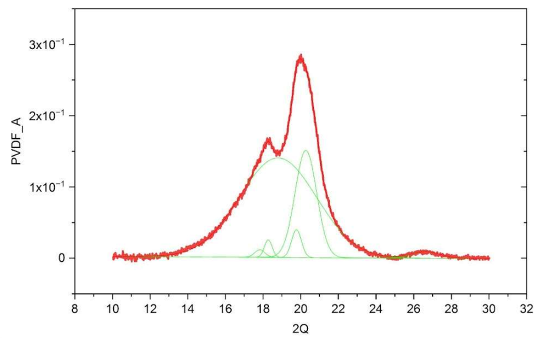

Fitting Results

Peak Index	Peak Type	Area Intg	FWHM	Max Height	Center Grvty	Area IntgP
1	Gauss	0.00717	0.63857	0.01055	17.82972	0.71989
2	Gauss	0.01262	0.48212	0.02458	18.27598	1.267
3	Gauss	0.02545	0.61035	0.03917	19.77615	2.5556
4	Gauss	0.01799	1.82793	0.00925	26.66491	1.8069
5	Gauss	0.21671	1.3537	0.15039	20.27328	21.7635
6	Gauss	0.71582	4.81948	0.13953	18.79675	71.88711

Peak Analysis

Data Set:[Book1]IntegResult1!O"PVDF/AU_R" Date:2024-09-19
BaseLine:Straight
Chi^2=5.91941E-06 Adj. R-Square=9.98750E-01 # of Data Points=978
SS=5.67672E-03 Degrees of Freedom=959

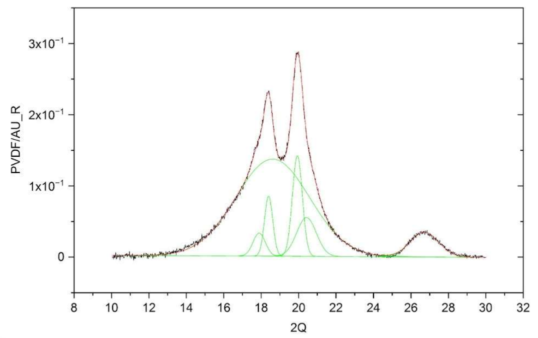

Fitting Results

Peak Index	Peak Type	Area Intg	FWHM	Max Height	Center Grvty	Area IntgP
1	Gauss	0.02648	0.77185	0.03223	17.89208	2.70536
2	Gauss	0.04633	0.51682	0.08422	18.39717	4.73437
3	Gauss	0.09426	0.62689	0.14125	19.93271	9.63107
4	Gauss	0.06631	1.79659	0.03467	26.72538	6.77496
5	Gauss	0.07184	1.23936	0.05446	20.42	7.34083
6	Gauss	0.67346	4.64268	0.13627	18.61598	68.8134

Peak Analysis

Data Set:[Book1]IntegResult1!P"PVDF/AU_A" Date:2024-09-19
BaseLine:Straight
Chi^2=8.74108E-06 Adj. R-Square=9.98257E-01 # of Data Points=978
SS=8.38270E-03 Degrees of Freedom=959

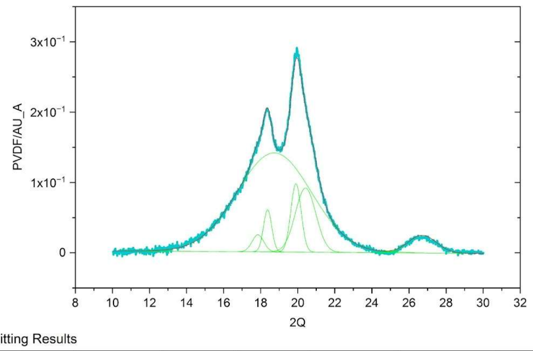

Fitting Results

Peak Index	Peak Type	Area Intg	FWHM	Max Height	Center Grvty	Area IntgP
1	Gauss	0.01894	0.73513	0.0242	17.85136	1.89601
2	Gauss	0.03333	0.52411	0.05975	18.37261	3.33697
3	Gauss	0.06724	0.64972	0.09722	19.90215	6.73083
4	Gauss	0.04754	1.77694	0.02513	26.71985	4.75895
5	Gauss	0.1261	1.30066	0.09108	20.42	12.62328
6	Gauss	0.70578	4.70969	0.14078	18.73372	70.65396

Peak Analysis

Data Set:[Book1]IntegResult1!M"PVDF/nHA_R" Date:2024-09-19
BaseLine:Straight
Chi^2=7.75444E-06 Adj. R-Square=9.98528E-01 # of Data Points=978
SS=7.35121E-03 Degrees of Freedom=948

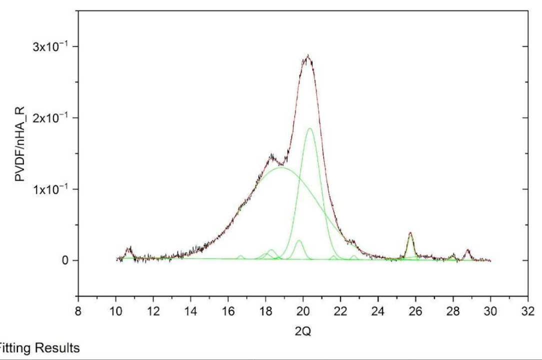

Fitting Results

Peak Index	Peak Type	Area Intg	FWHM	Max Height	Center Grvty	Area IntgP
1	Gauss	0	0	0	10.73359	0
2	Gauss	0.00433	0.54056	0.00752	18.01449	0.45046
3	Gauss	0.00762	0.54056	0.01324	18.30455	0.7928
4	Gauss	0.01537	0.54056	0.02671	19.78449	1.59912
5	Gauss	0.01087	1.94107	0.00526	26.36707	1.13064
6	Gauss	0.25803	1.31824	0.18389	20.36294	26.85068
7	Gauss	0.63655	4.66042	0.12832	18.83682	66.2388
8	Gauss	0.01279	0.35161	0.03418	25.72356	1.33111
9	Gauss	0.0017	0.24843	0.00643	27.97	0.17706
10	Gauss	0.00409	0.24928	0.01543	28.7748	0.42607
11	Gauss	0.00472	0.34011	0.01305	10.69	0.49153
12	Gauss	0.00122	0.23548	0.00488	16.67	0.12721
13	Gauss	7.27474E-4	0.23548	0.0029	18.64	0.0757
14	Gauss	0.0013	0.23548	0.00521	21.62	0.13577
15	Gauss	0.00166	0.25707	0.00608	22.71	0.17304

Peak Analysis

Data Set:[Book1]IntegResult1!N"PVDF/nHA_A" Date:2024-09-19
BaseLine:Straight
Chi^2=2.12245E-05 Adj. R-Square=9.96220E-01 # of Data Points=978
SS=2.01209E-02 Degrees of Freedom=948

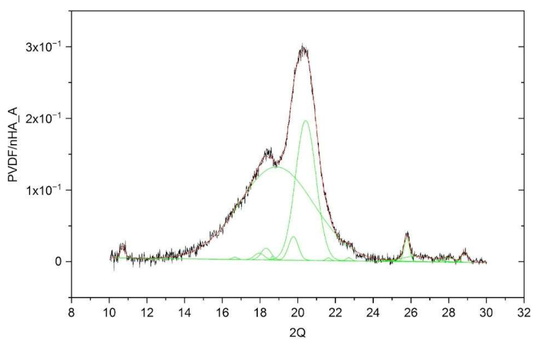

Fitting Results

Peak Index	Peak Type	Area Intg	FWHM	Max Height	Center Grvty	Area IntgP
1	Gauss	0	0	0	10.73359	0
2	Gauss	0.00602	0.6092	0.00928	17.96	0.60427
3	Gauss	0.01059	0.6092	0.01633	18.32	1.06351
4	Gauss	0.02136	0.6092	0.03294	19.77	2.14515
5	Gauss	0.0151	1.81619	0.00781	26.32239	1.51671
6	Gauss	0.26498	1.27556	0.19515	20.42109	26.60808
7	Gauss	0.64453	4.66197	0.12988	18.87439	64.72159
8	Gauss	0.01178	0.34081	0.03248	25.78038	1.18306
9	Gauss	0.00516	0.7828	0.0062	27.97	0.51859
10	Gauss	0.00593	0.4025	0.01383	28.85822	0.59502
11	Gauss	0.00587	0.32136	0.01715	10.70928	0.58923
12	Gauss	0.00113	0.32598	0.00324	16.67	0.11306
13	Gauss	6.7001E-4	0.12095	0.0052	18.64	0.06728
14	Gauss	0.0012	0.28507	0.00396	21.62	0.12067
15	Gauss	0.00153	0.31924	0.00451	22.71	0.1538

Peak Analysis

Data Set:[PVDFpellet10K]IntegResult1!K"I" Date:2024-09-19
BaseLine:Straight
Chi^2=8.23414E-05 Adj. R-Square=9.81940E-01 # of Data Points=980
SS=7.88007E-02 Degrees of Freedom=957

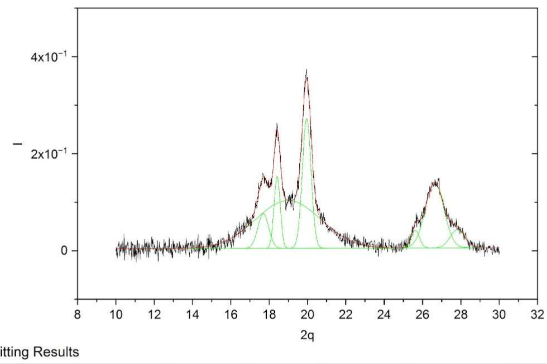

Fitting Results

Peak Index	Peak Type	Area Intg	FWHM	Max Height	Center Grvty	Area IntgP
1	Gauss	0.05054	0.66274	0.07164	17.69253	5.63255
2	Gauss	0.05949	0.37802	0.14784	18.41616	6.63009
3	Gauss	0.15173	0.53411	0.26688	19.95841	16.91027
4	Gauss	0.01936	0.49885	0.03645	25.62721	2.15707
5	Gauss	0.16329	1.13782	0.13482	26.63111	18.19852
6	Gauss	0.03577	0.89054	0.03774	27.82557	3.98688
7	Gauss	0.4171	3.97375	0.09861	18.98984	46.48463

Peak Analysis

Data Set:[HAP1exported]HAP_1_exported!L"Normalized Y4" Date:2024-09-19
BaseLine:Straight
Chi^2=5.44355E-05 Adj. R-Square=9.67981E-01 # of Data Points=980
SS=5.18770E-02 Degrees of Freedom=953

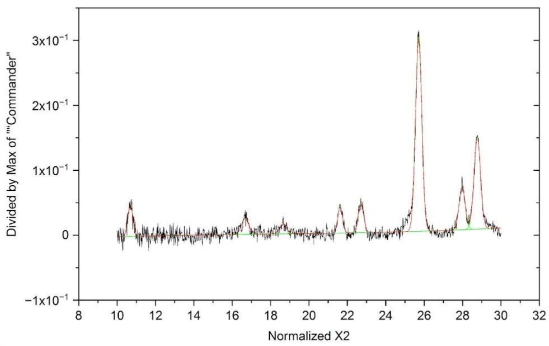

Fitting Results

Peak Index	Peak Type	Area Intg	FWHM	Max Height	Center Grvty	Area IntgP
1	Gauss	0.12256	0.38463	0.29936	25.71394	45.15891
2	Gauss	0.02592	0.36359	0.06698	27.97732	9.55189
3	Gauss	0.05995	0.39805	0.14148	28.76836	22.08809
4	Gauss	0.01592	0.30141	0.04964	10.69382	5.86757
5	Gauss	0.01168	0.43282	0.02536	16.67738	4.3046
6	Gauss	0.00695	0.43282	0.01509	18.64362	2.56138
7	Gauss	0.01251	0.28562	0.04116	21.62119	4.61064
8	Gauss	0.0159	0.32833	0.04548	22.70832	5.85692
